# Cost-Effective Bimetallic Catalysts for Green H_2_ Production in Anion Exchange Membrane Water Electrolyzers

**DOI:** 10.3390/nano15131042

**Published:** 2025-07-04

**Authors:** Sabrina Campagna Zignani, Marta Fazio, Mariarosaria Pascale, Chiara Alessandrello, Claudia Triolo, Maria Grazia Musolino, Saveria Santangelo

**Affiliations:** 1 Institute of Advanced Technologies for Energy (ITAE) of the National Research Council (CNR), 98126 Messina, Italy; sabrina.campagnazignani@cnr.it (S.C.Z.); fazio@itae.cnr.it (M.F.); pascale@itae.cnr.it (M.P.); 2 Department of Civil, Energy, Environmental and Materials Engineering (DICEAM), Mediterranean University of Reggio Calabria, 89122 Reggio Calabria, Italy; c.alessandrello@unirc.it (C.A.); claudia.triolo@unirc.it (C.T.); saveria.santangelo@unirc.it (S.S.); 3 National Reference Center for Electrochemical Energy Storage (GISEL), National Interuniversity Consortium for the Science and Technology of Materials (INSTM), 50121 Florence, Italy

**Keywords:** bimetallic electrocatalysts, green hydrogen, nanostructured electrodes, anion exchange membrane water electrolysis, alkaline electrolyzer

## Abstract

Green hydrogen production from water electrolysis (WE) is one of the most promising technologies to realize a decarbonized future and efficiently utilize intermittent renewable energy. Among the various WE technologies, the emerging anion exchange membrane (AEMWE) technology shows the greatest potential for producing green hydrogen at a competitive price. To achieve this goal, simple methods for the large-scale synthesis of efficient and low-cost electrocatalysts are needed. This paper proposes a very simple and scalable process for the synthesis of nanostructured NiCo- and NiFe-based electrode materials for a zero-gap AEMWE full cell. For the preparation of the cell anode, oxides with different Ni molar fractions (0.50 or 0.85) are synthesized by the sol–gel method, followed by calcination in air at different temperatures (400 or 800 °C). To fabricate the cell cathode, the oxides are reduced in a H_2_/Ar atmosphere. Electrochemical testing reveals that phase purity and average crystal size significantly influence cell performance. Highly pure and finely grained electrocatalysts yield higher current densities at lower overpotentials. The best performing membrane electrode assembly exhibits a current density of 1 A cm^−2^ at 2.15 V during a steady-state 150 h long stability test with 1 M KOH recirculating through the cell, the lowest series resistance at any cell potential (1.8 or 2.0 V), and the highest current density at the cut-off voltage (2.2 V) both at the beginning (1 A cm^−2^) and end of tests (1.78 A cm^−2^). The presented results pave the way to obtain, via simple and scalable techniques, cost-effective catalysts for the production of green hydrogen aimed at a wider market penetration by AEMWE.

## 1. Introduction

In sustainable energy societies, the development and use of clean, renewable and environmentally friendly energy is a necessary solution to address global energy demand and the rapid depletion of fossil fuels, as well as to solve the associated environmental pollution issues, such as environmental degradation and climate change [1,2,3]. Thanks to its pollution-free with only water and heat as combustion products, the high abundance and high energy density, hydrogen as an energy carrier is widely regarded as the most promising alternative to fossil fuels in the 21st century and will play a key role in the energy transition to achieve the goal of net zero CO_2_ emissions [4,5,6]. Among the various sustainable technologies for H_2_ production, electrochemical water splitting is one of the most efficient, feasible and clean options for generating high-purity hydrogen on a large scale, especially when it is powered by renewable energy sources [7,8,9].

Alkaline water electrolysis (AWE) and proton exchange membrane water electrolysis (PEMWE) are the most widely used technologies for low-temperature hydrogen production [7,10]. AWE, which works with a highly concentrated alkaline liquid electrolyte (typically an aqueous solution of 20–30 wt% KOH), a cheaper and porous diaphragm separator, and non-noble metals as electrocatalysts, represents a cost-effective and industry-proven technology [11]. However, AWE suffers from some drawbacks, such as handling a highly caustic electrolyte and low current densities. In addition, the diaphragm, failing to completely prevent the cross-over of the produced gases from one half-cell to the other, leads to lower energy efficiency and safety issues [10,12]. PEMWE technology, which uses an ultrathin polymer membrane as a solid electrolyte with high proton conductivity and a pure water feed, features a compact device design, higher current density, higher efficiency, and higher hydrogen purity than AWE [13]. Nonetheless, the presence of a harsh and acidic environment, due to the proton exchange membrane, requires the use of specific and expensive corrosion-resistant materials for electrolyzer components. Platinum group metals (PGMs)-based catalysts, like Pt for the cathode and IrO_2_/RuO_2_ for the anode, Nafion-type membrane, titanium-based current collectors, and bipolar plates are commonly used [14,15]. Therefore, high investment costs continue to be a major bottleneck for the large-scale application of PEMWE technology.

Recently, anion exchange membrane water electrolysis (AEMWE) has emerged as an appealing process for green hydrogen production and a potential solution to the shortcomings of both AWE and PEMWE technologies [10,16,17,18]. AEMWE combines the cost-effectiveness of AWE with the advantages of PEMWE, such as hydrogen purity and high current density. Similar to PEMWE, this technology employs a thin anion exchange membrane (AEM) as the solid polymer electrolyte and a zero-gap cell configuration, enabling a compact cell design with lower ohmic resistance, higher operating current densities, and, by efficiently separating hydrogen, high-purity hydrogen production. Furthermore, since the AEM conducts hydroxide ions, an alkaline working environment is present as in the AWE, which allows the use of non-precious transition metals and their derivatives as electrocatalysts and inexpensive materials for membranes and bipolar plates, improving economic feasibility [10,16,17,18,19,20,21].

Overall electrochemical water splitting process occurs through two half-cell reactions: hydrogen evolution reaction (HER) at the cathode and oxygen evolution reaction (OER) at the anode. Under standard conditions, a theoretical thermodynamic potential of 1.23 V is required to drive the water splitting process, corresponding to an energy consumption of ∆G = 237.1 kJ mol^−1^ [19,22]. However, a higher cell voltage (overpotential) is necessary to overcome the intrinsic energy barriers due to the sluggish kinetics of the HER and OER reactions, especially for the OER involving a 4-electron transfer and more intermediates, and the ohmic resistance of the electrolyte and cell components [10,18,23,24]. Overpotentials result in high energy consumption and low energy conversion efficiency. Therefore, the development of highly active, low-cost, sustainable, and durable catalysts is crucial to simultaneously accelerate the kinetics of HER and OER and efficiently reduce the overpotentials, improving the performance of electrochemical water splitting.

Over the past decade, first-row transition metal-based compounds and, in particular, Ni-based materials, including alloys [25,26], oxides [27,28], layered double hydroxide [29,30], chalcogenides [31,32], phosphides [33,34], and nitrides [29,35], have been developed as promising OER and HER catalysts for the AEMWE, due to their stability and corrosion resistance in an alkaline environment. However, most of the reported works are limited to half-cell studies. Furthermore, catalysts with outstanding activity towards OER often show poor HER activity, due to the different mechanisms. As a result, Pt-based benchmark catalysts continue to be the primary cathodic material in an AEMWE single-cell configuration to achieve low overpotentials and high current densities. Thus, the design of effective and stable non-PGM bifunctional catalysts for both OER and HER reactions in an alkaline environment remains an urgent challenge. The use of the same material as a catalyst at both the anode and the cathode leads to a simplification of the electrolysis process and a significant reduction in the overall cost, because, for example, different equipment and methodologies are not required to produce different electrocatalysts. Although some progress has been made, very few studies that meet the above requirements and show long-term operational lifetime are reported on the overall water splitting in AEMWE [18,22].

Considering the above context, the focus of this work is to develop electrocatalysts through a very simple and scalable synthesis method and to study their electrocatalytic activity and long-term stability towards water electrolysis in an AEMWE single-cell configuration. It is widely recognized that the combination of non-precious metals, such as nickel/cobalt and nickel/iron, in oxides/hydroxides/oxyhydroxides leads to sustainable electrocatalysts with interesting OER properties. This is often understood as the effect of the synergistic interaction between the two metals, potentially resulting in enhanced conductivity and the formation of more active sites. Furthermore, the structure and catalytic activity of bimetallic catalysts can be influenced by the ratio of the two metals [36,37]. Here, nanostructured NiCo- and NiFe-oxides, with different concentrations of the two metals (50:50 or 85:15), are produced by the sol–gel method followed by calcination in air at two different temperatures (400 or 800 °C) to investigate the effect of crystal size and crystallization degree. The as-prepared and H_2_/Ar-reduced binary oxides are used as electrocatalysts to fabricate the anode and cathode, respectively. The electrodes are then assembled in a zero-gap AEMWE full cell, in combination with a commercial polymeric anion exchange membrane (Fumatech^®^ FAA3-50) and a commercial ionomer (ION FAA-3-SOLUTION). Reference monometallic (nickel) oxide and hydroxide are also prepared and tested. Electrocatalysts are thoroughly characterized by complementary analysis techniques to investigate the structural factors playing a key role in their electrocatalytic performance.

## 2. Materials and Methods

### 2.1. Synthesis of OER Electrocatalysts

Nanostructured bimetallic oxides to be used as OER electrocatalysts at the cell anode were prepared via the sol–gel (SG) method and subsequent calcination, following the procedure described in detail in a previous paper [38]. Nickel (II) acetate tetrahydrate (purity > 98%, CAS No. 6018-89-9, Merck), cobalt (II) acetate tetrahydrate (purity > 98%, CAS No. 6147-53-1, Merck), and iron acetate (purity > 95%, CAS No. 3094-87-9, Merck) were used as Ni, Co, and Fe sources, respectively. Stoichiometric amounts of these salts (Table 1) were dissolved in 60 g of water, one at a time (Figure S1a). After magnetic stirring at 250 rpm for 30 min, 6 g of monohydrate citric acid (purity 98%; CAS No. 5949-29-1, Merck) was added as a complexing agent, and the resulting mixture was further stirred at 350 rpm for 2 h at 70 °C to form a gel. The as-obtained gel was dried at 80 °C overnight and, subsequently, calcined in static air in a muffle furnace (Figure S1b). Temperature was increased at a rate of 10 °C min^−1^ and kept constant for 2 h at the selected value (*T*_C_, Table 1). Calcination was followed by rapid cooling down to room temperature (RT) out of the furnace to generate defects on the oxide surface [38,39,40] and enhance porosity and oxygen deficiency [41].

A reference monometallic (nickel) oxide was further prepared via the same procedure (Table 1). In addition, a reference nickel hydroxide sample (coded as NiH100) was synthesized from nickel (II) nitrate hexahydrate (purity > 99%, CAS No. 13138-45-9, Aldrich) via the co-precipitation (CP) method. Nickel nitrate hexahydrate was dissolved in ultrapure distilled water at 60 °C to form a suspension that was neutralized at pH 9 with a 1 M NaOH solution. The suspension was stirred and kept at 60 °C and pH 9 for 4 h to promote the precipitation of the compound. The precipitate was then filtered, washed with heated ultrapure water, and dried at 80 °C overnight. The scheme of electrocatalyst preparation is provided in Figure S2.

### 2.2. Synthesis of HER Electrocatalysts

A portion of the as-prepared oxides was utilized to produce HER electrocatalysts for the cell cathode (Figure S3a). Oxide powders were reduced in 5% H_2_/Ar atmosphere for 30 min (Figure S3b) to form reduced bimetallic compounds (Figure S3c). The reduction temperatures (*T*_R_) of each material, reported in Table S1, were determined by means of the temperature programmed reduction (TPR) analysis (Figure S4), carried out on an AMI-300 instrument.

### 2.3. Electrocatalysts Characterization

All electrocatalysts were characterized through scanning electron microscopy (SEM) and energy-dispersive X-ray (EDX) analysis using an SEM-FEG-UHR microscope (Thermo Fisher) equipped with a FIB (focused ion beam) column and STEM (scanning transmission electron microscopy) detectors. The instrument was operated in the range 2–15 kV, and the EDX probe was used to determine the bulk elemental composition of the samples.

The oxide phases formed under calcination in OER catalysts and the metallic species obtained by reduction in HER catalysts were identified by X-ray diffraction (XRD). The analysis was carried out with a Bruker D2 diffractometer using Ni β-filtered Cu-K_α_ radiation source (λ = 0.1541 nm). As usual [42], to determine the phase composition of the electrocatalysts, diffractograms were analyzed by the Rietveld method using Maud 2.992 software and the isotropic model for all samples except for NiH100. For the latter sample, the Popa model was used [43] in order to more accurately describe the anisotropic behavior of the crystallites, which is well documented in the literature [44,45].

The spatial homogeneity of the oxides was assessed by measuring Raman scattering from various random positions on each specimen. For this purpose, an NTEGRA—Spectra SPM NT-MDT confocal microscope coupled to a solid-state laser operating at 532 nm was used. Measurements were carried out in air at RT by using a low laser power (250 μW at the sample surface) to prevent local heating. The scattered light from the sample was collected by a 100X Mitutoyo objective (NA = 0.75), dispersed by an 1800 lines mm^−1^ grating, and detected by a cooled ANDOR iDus CCD Camera. Since the surface area of the region probed by micro-Raman spectroscopy (MRS, <0.6 μm^2^) is smaller than that probed by XRD, to assess spatial homogeneity of the oxides, spectra were recorded from random positions on each specimen and then averaged to infer reliable information on the entire sample.

The surface chemical composition of the samples and chemical environment were evaluated by X-ray photoelectron spectroscopy (XPS). Spectra were recorded using a Physical Electronics GMBH PHI 5800-01 spectrometer (Physical Electronics GmbH, Munich, Germany), equipped with a monochromatic Al-K_α_ source (1486.6 eV) with a power beam of 300 W. XPS data were interpreted using the online library of oxidation states implemented in PHI MultiPak 6.1 software (Chanhassen, MN, USA, 1999) and the PHI Handbook of X-ray photoelectron spectroscopy.

### 2.4. Anionic Electrolyte

A commercial Fumatech^®^ FAA3-50 membrane was used as a solid polymer electrolyte in AEM electrolysis. The selected thickness of the membrane (50 μm) was chosen from a compromise between low cross-over, low area-specific resistance, and suitable mechanical stability requirements. FAA3-50 membranes contain ammonium functional groups, allowing the exchange of hydroxyl ions, characterized by a lower mobility compared to protons, from the cathode compartment to the anode, where the OER takes place. By doing so, the recombination of the reaction products can be avoided while ions can percolate in the device as needed to complete the electrochemical process. Before use, the membrane needs to be activated by treating it with a 3 M NaCl solution for 72 h in order to obtain the membrane in Cl^−^ form. This step is necessary to facilitate the exchange between Cl^−^ and OH^−^ ions before cell assembly. A commercial ionomer (ION FAA-3-SOLUTION) from Fumatech^®^ was utilized as a binding agent during ink preparation in order to improve the adhesion of the electrocatalyst powder to the surface support.

### 2.5. Membrane Electrode Assembly and Electrochemical Studies

To prepare the membrane electrode assemblies (MEAs), the first step was to mix together the anionic ionomer, electrocatalyst powder, and solvent to create the anode and cathode inks for spray deposition onto specific supports. The ionomer, used to prepare the inks, was a commercial ION FAA-3-SOLUTION (10 %wt) from Fumatech^®^. Spray coating was used to deposit the catalysts on the support layers after dispersing them in ethanol and sonicating them for 30 min (Figure S5). To facilitate ethanol evaporation, a heated plate was used. A correct distance was maintained between the airbrush and the support to avoid ink penetration. Figure S6 shows a typical anode and cathode, Fumatech^®^ membrane, and MEA with a 5 cm^2^ active area used for the electrochemical test.

The anode inks, based on catalysts in their oxidized form (Table 1), were deposited on a nickel felt (Bekaert). Gas diffusion layers (GDLs) based on carbon paper (39BB, SIGRACET) were used as a support to deposit the cathodic inks, based on catalysts in their reduced form (Table S1). The mass loads were about 2.5 mg cm^−2^ and 3 mg cm^−2^ for the anode and cathode electrocatalysts, respectively.

The anode and cathode compartments were separated by a commercial Fumatech^®^ anionic exchange membrane (50 μm thickness) in the OH^−^ form. Before the assembly, the membrane and the electrodes, containing the ionomer, were exchanged for 24 h with a solution containing hydroxide ions in order to activate the components.

Figure S7 displays the operation scheme for the AEM electrolysis cell and its functional components. A cold assembly procedure was followed to prepare the MEAs to avoid any undesired degradation of the membrane, as observed during the conventional hot-pressing lamination process. The MEAs were assembled in a single-cell housing made of nickel plates characterized by a serpentine flow field channel that matches the active area of 5 cm^2^. Teflon^®^ gaskets were used to seal the cell and avoid any leakage of the electrolyte solution. Cell compression was 2.5 N m per tie rod, and this tightening for the cell can ensure that the electrodes and the membrane are in direct contact like a real sandwich. The single cell was tested at 50 °C and under atmospheric pressure. During all experiments, the anode compartment was fed with 1 M KOH solution at a flow rate of 5 mL min^−1^ using a peristaltic pump. The cell was maintained at the same temperature using heating mats applied to the external plates of the cell.

### 2.6. Electrochemical Studies

The system was characterized electrochemically to study the performance of the MEAs in terms of reaction rates at different cell potentials. To investigate the electrochemical behavior of each MEA, galvanostatic polarization curves (cell voltage versus current density) were recorded, and galvanostatic durability tests (cell voltage versus time) and electrochemical impedance spectroscopy (EIS) analyses were performed. Polarization curves and chrono-potentiometric curves were carried out with a Keithley power supply system (Tektronic), a PGSTAT Autolab 302 Potentiostat/Galvanostat equipped with a booster of 20 A (Metrohm), and a Frequency Response Analyzer (FRA) was used for EIS analyses. EIS measurements were performed under potentiostatic control in a frequency range between 1 MHz and 10 mHz by frequency sweeping in a single sine mode and acquiring ten points per decade. The amplitude of the sinusoidal excitation signal was 0.01 V r.m.s. The series resistance was determined from the high frequency intercept on the real axis in the Nyquist plot, while the polarization resistance was estimated as the difference between the extrapolated low frequency intercept and the high frequency intercept on the real axis [30].

## 3. Results and Discussion

### 3.1. OER Electrocatalysts

#### 3.1.1. Morphology

**Figure 1:**
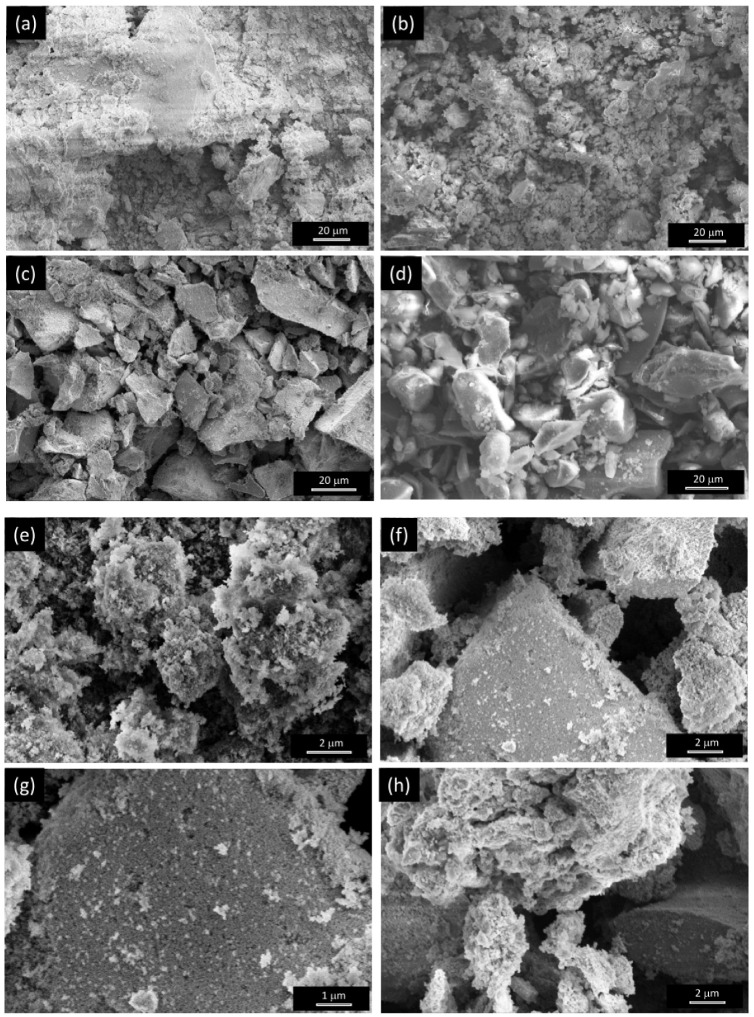
Morphology of the oxides as resulting from SEM analysis. The shown micrographs refer to samples (**a**,**e**) NiH100, (**b**) Ni100, (**c**,**f**,**g**) Ni85Co15_400, and (**d**,**h**) Ni85Fe15_400.

The morphology and composition of all electrocatalysts were investigated by SEM and SEM/EDX. Figure 1 shows some representative SEM images of the OER electrocatalysts investigated. Lower magnification micrographs are shown in Figure S8a–c. The results of elemental analysis by SEM/EDX are reported in Figure S8d–f. Regardless of the synthesis method, large agglomerates of small particles are formed, as proven by higher magnification images (Figure 1g). In the case of the sample (NiH100) prepared via the CP method, the particle aggregates look more compact.

The results of SEM/EDX analysis demonstrate that the organic components of the metal precursors are completely removed during calcination, with the formation of inorganic oxides. The identification of their phase(s) was carried out via XRD analysis.

#### 3.1.2. Phase(s) and Average Crystallite Size of the Oxides

**Figure 2:**
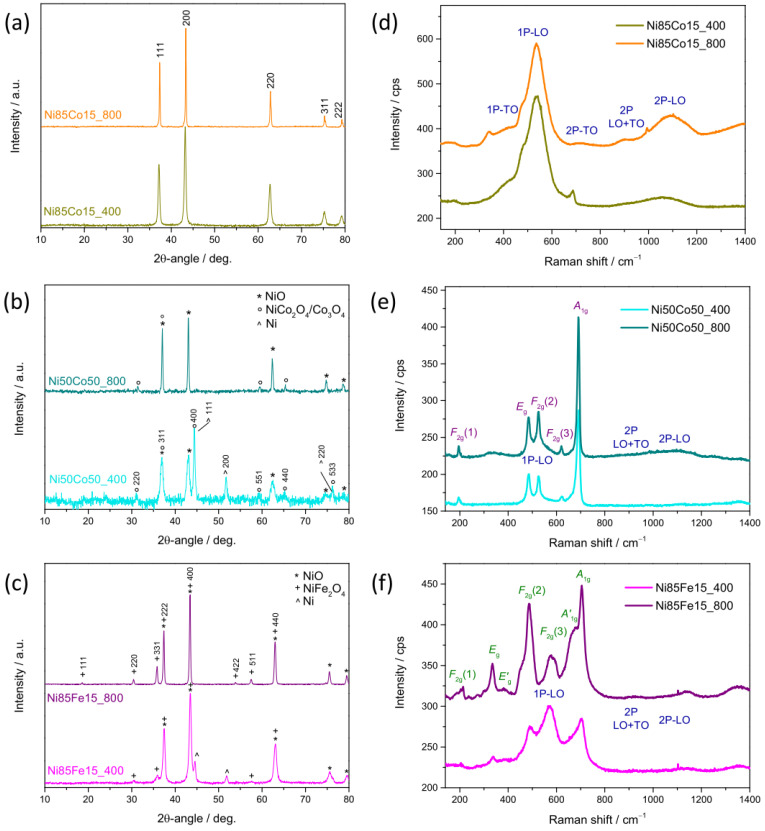
Results of (**a**–**c**) XRD and (**d**–**f**) MRS analyses on bimetallic oxides. The shown diffractograms refer to (**a**) Ni85Co15, (**b**) Ni50Co50, and (**c**) Ni85Fe15 electrocatalysts. Micro-Raman spectra refer to (**d**) Ni85Co15, (**e**) Ni50Co50, and (**f**) Ni85Fe15 electrocatalysts.

Figure S9 displays the diffractograms of the reference monometallic samples; the XRD patterns of bimetallic oxides are shown in Figure 2a–c. All diffraction peaks detected in the reference NiH100 sample (top of Figure S9b) can be indexed to the β-phase of nickel hydroxide [46,47]. This evidences the formation of a single phase. Similarly, only the reflections from the (111), (200), (220), (311), and (222) crystallographic planes of cubic NiO with rock-salt (RS) structure at ca. 37.30°, 43.34°, 62.89°, 75.45°, and 79.40° 2θ-angles, respectively, are detected in the monometallic oxide (Ni100) prepared by SG (top of Figure S9a) [48,49,50].

In the case of bimetallic oxides (Figure 2a–c), XRD analysis reveals that the phase purity of the oxide is influenced by the metal pair and their molar fractions and, in some cases, also by the temperature at which the oxidative heat treatment is operated. A single RS phase forms at any *T*_C_ in Ni85Co15 oxides (Figure 2a) [48,49,50]. Since the radius of the Co^2+^ ion is similar to that of the Ni^2+^ ion (58 pm against 55 pm for tetrahedral coordination, respectively [51]), the substitution of a small amount (0.15 molar fraction) of Ni with Co in the cubic RS lattice easily takes place and results a uniform dispersion of Co into the NiO matrix [52] with no secondary phase formation.

As the molar fraction of Co rises to 0.50, additional reflections appear in the XRD patterns of NiCo-based oxides (Figure 2b). This finding suggests that the segregation of a portion of Co/Ni in different phase(s) takes place. At higher *T*_C_ (Ni50Co50_800), the reflections from the (220), (331), (511), and (440) crystallographic planes of a phase with spinel (SP) structure are detected at ca. 31.38°, 37.60°, 59.55°, and 65.37° 2θ-angles, along with the signals arising from the RS lattice (top of Figure 2b). Thus, the increase in the Co molar fraction up to 0.50 promotes the formation of a biphasic oxide, in agreement with other literature reports [53,54]. Besides NiO, cobalt (II, III) oxide (Co_3_O_4_) and/or nickel cobaltite (NiCo_2_O_4_) formed. In Ni50Co50_400, the reflections from Ni/α-Co metals with cubic structure are further clearly visible in the diffraction pattern (bottom of Figure 2b) [49,50,55], revealing that the oxidation upon thermal treatment is incomplete, and as a result, a three-phase material is formed.

NiFe-based oxides (Figure 2c) behave similarly to the equimolar NiCo-based oxides. In the diffractogram of Ni85Fe15_800, in addition to the reflections from the cubic RS NiO lattice, those originating from the crystallographic planes of a SP-structured phase appear (top of Figure 2c), indicating the formation of a biphasic oxide, in line with literature reports [56]. Nickel ferrite (NiFe_2_O_4_) forms together with cubic NiO. At lower *T*_C_ (Ni85Fe15_400), the reflections from Ni/Co metals are also visible (bottom of Figure 2c) [49,50,55].

Additional information on the material structure and composition was inferred by carrying out Rietveld refinements from XRD data (Figure S10). The main results obtained are summarized in Table 2 and Figure S11; further details are reported in Table S2. The formation of a pure single-phase RS structure is confirmed in the case of monometallic Ni100 oxide and bimetallic Ni85Co15 oxides, regardless of their calcination conditions. In these materials, the average crystallite size (*d*) increases in the order Ni100 < Ni85Co15_400 << Ni85Co15_800 (Table 2).

In the remaining bimetallic oxides, two or three phases coexist, depending on *T*_C_. RS is always the primary phase. Nonetheless, at 800 °C, its amount ranges between 72 wt% (in Ni50Co50_800) and 81 wt% (in Ni85Fe15_800); whereas, at lower *T*_C_, it reduces to 77 wt% in Ni85Fe15_400 and drops down to 47 wt% in Ni50Co50_400. The comparison between oxides with the same nominal composition reveals that more oxidized species are formed at higher *T*_C_, as expected [41]. In fact, in Ni50Co50_800, a larger amount of Co_3_O_4_/NiCo_2_O_4_ is present compared to Ni50Co50_400 (27.8 against 22.6 wt%); analogously, in Ni85Fe15_800, NiFe_2_O_4_ is more abundant than in Ni85Fe15_400 (18.6 against 10.4 wt%). Furthermore, by comparing the composition of bimetallic materials calcined at 400 °C, it emerges that the metal/alloy component, absent in Ni85Co15_400, increases from 12.4 wt% in Ni85Fe15_400 up to 30.6 wt% in Ni50Co50_400. Finally, larger *d*-values are obtained upon calcination at 800 °C due to sintering effects (Table 2). At 400 °C, the presence of Co/a larger molar fraction of Co seems to favor the development of larger crystallites (compare *d*-values of Ni85Fe15_400 and Ni85Co 15_400, and of Ni85Co 15_400 and Ni50Co 50_400).

As a general behavior, some small shifts are observed when comparing the patterns of the samples with the same nominal composition calcined at different temperatures (Figure 2). Such shifts can be ascribed to different levels of microstrain affecting the lattice [42]. As shown in Table S2, for each oxide phase, the local distortions of the crystal structure and microstrain generally decrease in better crystallized samples (calcined at higher temperatures).

#### 3.1.3. Spatial Uniformity and Crystallization Degree of the Oxides

Spatial uniformity of the OER electrocatalysts was assessed by measuring Raman scattering from random positions on each specimen (Figures S12 and S13). Except for sample Ni85Fe15_400 (Figure S13c), where even remarkable dissimilarities are observed between spectral profiles recorded at different locations, no significant differences are visible in the remaining samples, which proves the spatial uniformity of the oxide phase(s) formed.

Figure 2d–f displays the averaged micro-Raman spectra. Cubic RS structure belongs to the *Fm3m* space group. Two one-phonon (1P) modes and three two-phonon (2P) modes are predicted for this group [57,58]. The former modes comprise one transverse optical (TO) and one longitudinal optical (LO) mode; the latter includes the overtones of TO and LO modes and a combination band. 1P-TO and 1P-LO modes are symmetry-forbidden in a perfect cubic lattice [57,59]. Their detection (at ~410 and ~540 cm^−1^, respectively) in Ni100 (Figure S12) and Ni85Co15 oxides (Figure 2d) is indicative of the presence of lattice distortion, defect-induced disorder, and surface effects [57,59]. Also, the 2P-TO (at ~730 cm^−1^), 2P-TO+LO (at~905 cm^−1^), and 2P-LO (at ~1080 cm^−1^) are clearly visible in the higher frequency region of the spectra. These findings confirm the indications provided by XRD analysis. Moreover, in Ni85Co15_800 oxide, featured by larger-sized crystallites, the relative (to 2P-LO) intensity of the disorder-activated 1P-LO mode is weaker than in Ni85Co15_400, which indicates a reduced extent of structural disorder [57].

Spinel-structured phase belongs to the *Fd3m* space group. Based on the factor group analysis, five normal Raman-active vibration modes (*A*_1g_ + *E*_g_ + 3*F*_2g_) are predicted for this space group [60,61,62]. Both their positions and their relative intensities remarkably vary within the spinel family. In Ni50Co50 oxides (Figure 2e), at any *T*_C_, the sharp *A*_1g_ mode at 691 cm^−1^ dominates the spectra. The weaker *F*_2g_(1), *E*_g_, *F*_2g_(2), and *F*_2g_(3) modes are detected at frequencies (195, 483, 525, and 620 cm^−1^, respectively) very close to those reported for crystalline Co_3_O_4_ [62,63,64,65]. In the spectrum of Ni50Co50_800, the narrow *E*_g_ and *F*_2g_(2) peaks originating from the SP-structured component of the oxide are superimposed to the broad band arising from the 1P-LO mode of the defective RS phase; in the high-frequency region, the very weak 2P modes of this phase are observed, in agreement with the evidences emerged from XRD analysis.

In the spectra of Ni85Fe15 oxides (Figure 2f), along with the sharp peaks associated to the normal Raman vibration modes of the SP-structured NiFe_2_O_4_ phase at 214 (*F*_2g_(1)), 335 (*E*_g_), 486 (*F*_2g_(2)), 578 (*F*_2g_(3)), and 704 cm^−1^ (*A*_1g_) [60,66,67], two shoulders appear on the higher-frequency side of the *E*_g_ band (at 385 cm^−1^) and on the lower-frequency side of the *A*_1g_ band (at 674 cm^−1^). As known [66], nickel ferrite crystallizes in the inverse SP structure, with the tetrahedral 8*a* sites fully occupied by Fe^3+^ cations and the octahedral 16*d* sites occupied by both Ni^2+^ and Fe^3+^ cations. Thus, the additional inversion-induced spectral features (*E’*_g_ at 385 cm^−1^ and *A’*_1g_ at 674 cm^−1^) are due to distortions in the lattice caused by a distribution of distances between Ni and Fe with oxygen [67]. In the lower-frequency spectral region, the modes of the NiFe_2_O_4_ component of the Ni85Fe15 oxides are superimposed on the weaker ones originating from the defective RS-structured component.

### 3.2. HER Electrocatalysts

HER electrocatalysts for the cell cathode were prepared via reduction of the as-produced oxide powders at *T*_R_ (Table S1) in 5% H_2_/Ar atmosphere for 30 min. Figure S4 shows the TPR spectra. A single H_2_ consumption peak dominates the spectra of reference Ni100 and NiH100 samples (Figure S4a); the peak is located at 450 and 300 °C, respectively, indicating that in NiH100, the reduction of Ni^2+^ to Ni^0^ is easier. As a general result, in the bimetallic oxides calcined at higher temperatures (better crystallized) [41], the reduction of metal cations takes place at higher temperatures (compare the highest H_2_ consumption peaks in Figure S4b–d). In these materials, the lower-*T* H_2_ consumption peaks can be ascribed to the reduction of Ni, while the higher-*T* ones can be associated with the multi-step reduction of Co and Fe [68].

#### 3.2.1. Morphology

**Figure 3:**
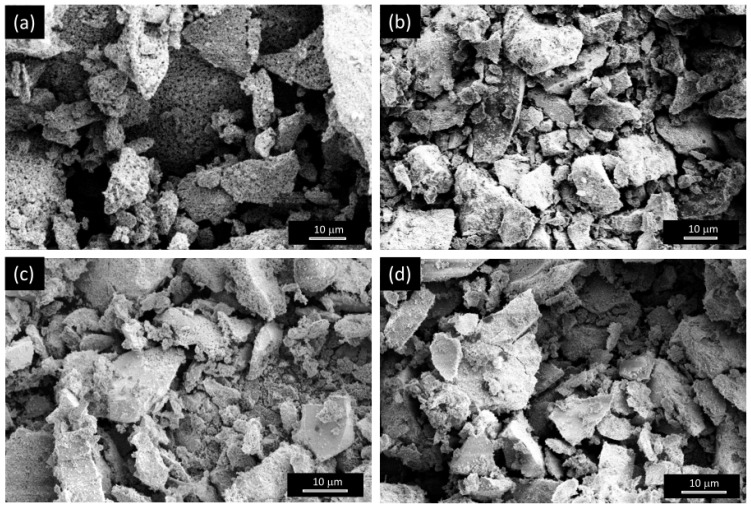

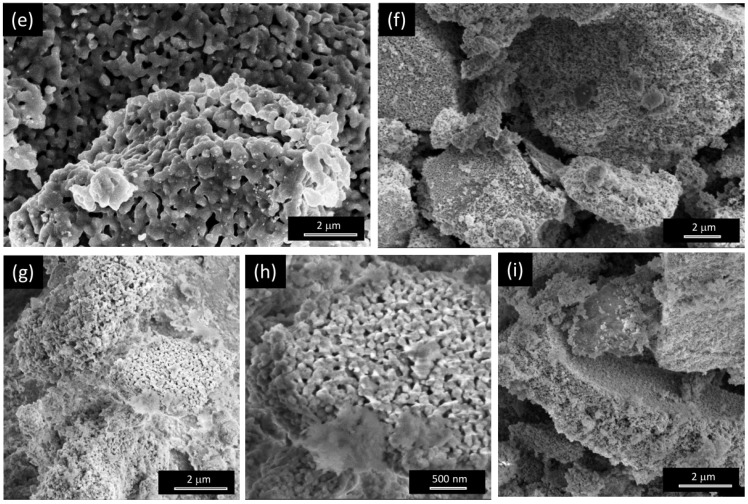
Morphology of the samples after reduction, as resulting from SEM analysis. The shown images refer to samples (**a**,**e**) NiH100R, (**b**,**f**) Ni100R, (**c**,**g**,**h**) Ni85Co15_400R, and (**d**,**i**) Ni85Fe15_400R.

SEM analysis (Figure 3 and Figure S14) reveals that the reduction process does not produces substantial changes in the morphology of the electrocatalysts prepared by the SG method (Figure 3b–d,f–i), while that prepared by via the CP method (Figure 3a,e) look less compact after the exposure to the H_2_/Ar atmosphere. In any case, the macro-aggregates are still made up of small particles, as clearly visible in Figure 3f–i.

Elemental analysis by SEM/EDX shows total or substantial removal of oxygen (compare Figures S8 and S15), confirming the successful reduction of materials in the oxidized form.

#### 3.2.2. Crystalline Phase(s) and Average Crystallite Size

The phases formed from the pristine oxides upon reduction in H_2_/Ar atmosphere at a proper temperature (Table S1) were investigated by XRD. Only the reflections at ca. 44.38°, 51.73°, and 76.26° 2θ-angles from the crystallographic planes of face-centered cubic (fcc) nickel [49,50] are detected in the two reference samples Ni100R and NiH100R (bottom of Figure S9a,b, respectively), confirming the full reduction of the pristine NiO and β-Ni(OH)_2_ phases, respectively.

**Figure 4:**
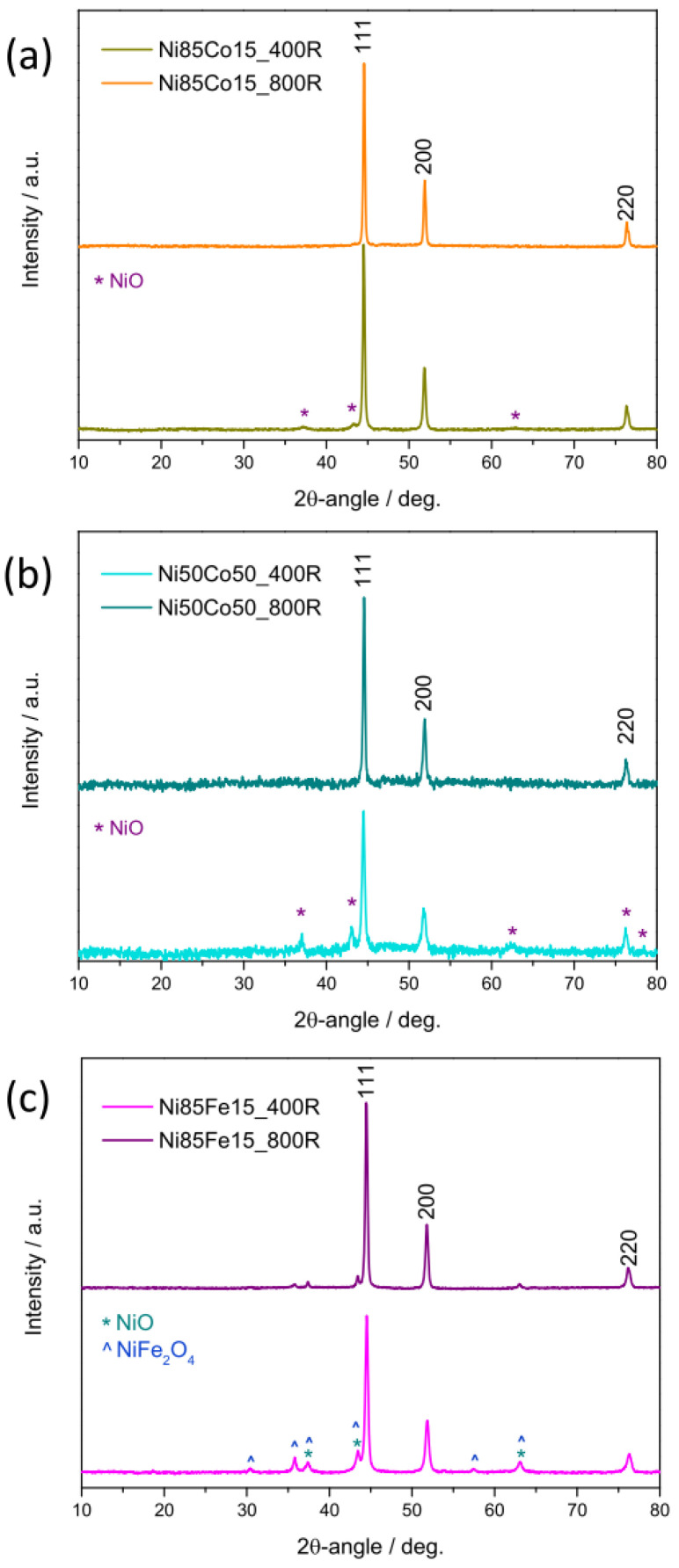
XRD patterns of HER electrocatalysts. The shown data refer to samples (**a**) Ni85Co15R, (**b**) Ni50Co50R, and (**c**) Ni85Fe15R.

The diffraction patterns recorded on the materials obtained by reducing bimetallic oxides are shown in Figure 4. XRD analysis demonstrates that obtaining a single bimetallic phase (alloy) strongly depends not only on the composition of the pristine oxide but also on its preparation conditions. Only the reflections from the (111), (200), and (220) crystallographic planes of Ni/α-Co metals with fcc structure [50,55] are detected in Ni85Co15_800R (top of Figure 4a) and Ni50Co50_800R electrocatalysts (top of Figure 4b), indicating the formation of a pure NiCo alloy.

Conversely, signals arising from the NiO component of the pristine oxides are still visible in the diffractograms of Ni85Co15_400R (bottom of Figure 4a) and Ni50Co50_400R (bottom of Figure 4b), indicating that the reduction is not complete, in agreement with the indications provided by elemental analysis by SEM/EDX. Thus, these electrocatalysts are biphasic in nature: Besides the NiCo alloy, they contain NiO as a secondary phase. Moreover, the stronger relative intensity of the NiO diffraction peaks in Ni50Co50_400R indicates the lower phase purity of this catalyst.

In Ni85Fe15_400R (bottom of Figure 4c), reflections from crystallographic planes of NiO and NiFe_2_O_4_ are detected, along with those from the fcc structure typical of Ni-rich NiFe alloy [69]. Thus, the electrocatalyst consists of three phases and has poor phase purity. A higher phase purity degree pertains to Ni50Co50_800R (top of Figure 4c), in whose diffractogram signals from the unreduced pristine oxides are extremely weak and hardly visible.

In order to get further information on the material structure and composition, Rietveld refinements from XRD data were carried out (Figure S16). Table 3 summarizes the main results obtained. Further details can be found in Table S3. Rietveld analysis confirms that the highest phase purity degree pertains to reference Ni100R and NiH100R electrocatalysts (containing Ni metal only) and to bimetallic Ni85Co15_800R and Ni50Co50_800R electrocatalysts (consisting of a pure NiCo alloy). In the former, the average size of Ni metal crystallites is around 61 nm. In Ni85Co15_800R and Ni50Co50_800R electrocatalysts, very large NiCo alloy crystallites are obtained (89 and 139 nm, respectively).

In all remaining HER electrocatalysts, the reduction of the pristine oxides is not complete. The phase purity worsens in the order Ni85Co15_400R > Ni85Fe15_800R > Ni50Co50_400R > Ni85Fe15_400R, with the relative amount of residual oxide phase(s) ranging from 6.7 wt% in Ni85Co15_400R to 28.3 wt% in Ni85Fe15_400R. The average size of the crystallites is 39 nm in both Ni85Fe15_400R and N50Co50_400R and larger in the remaining impure alloys (Table 3).

#### 3.2.3. Nanomaterial Selection

Figure S17a,b summarize the properties of the produced electrode materials in terms of phase purity and mean crystal size. Crystal size seems to be particularly critical for the anode electrocatalysts: Smaller-sized crystals, featured by larger exposed surface available for reaction and greater amounts of grain boundaries, generally exhibit a higher density of reactive surface defects [38,41,70], which is beneficial to OER [41]. Phase purity seems to play a pivotal role, too. Actually, very recently, better electrochemical performance has been reported for pure single-phase multi-component oxides [71]. Accordingly, further physicochemical characterization (XPS analysis) and electrochemical tests are performed on a restricted set of five pairs of samples, selected by choosing the best compromise between high phase purity and small average crystal size.

#### 3.2.4. Species on the Surface of OER Electrocatalysts

**Figure 5:**
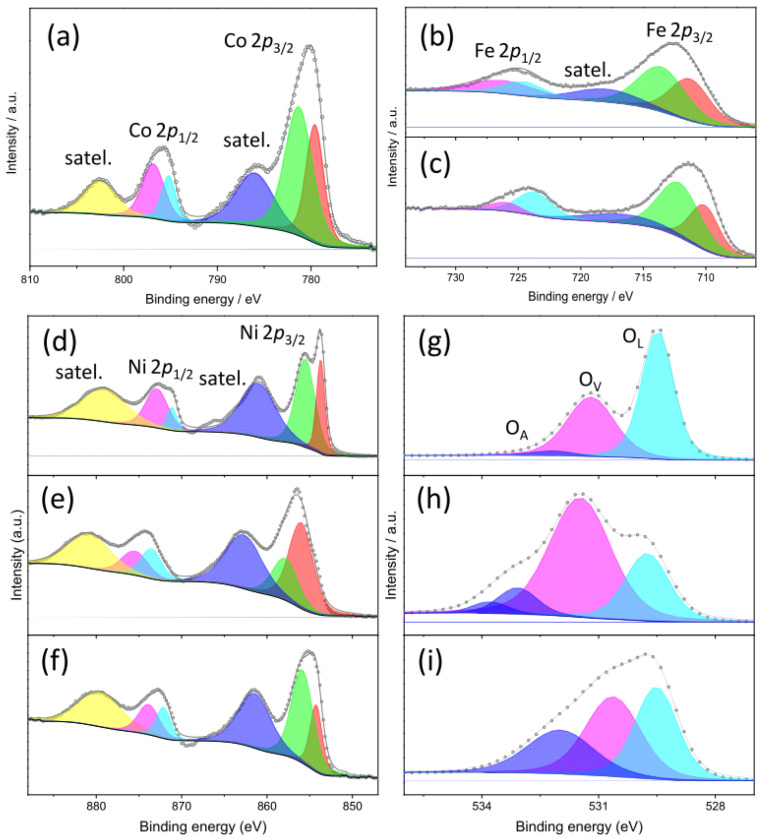
High-resolution XPS curves and fitting in the regions of (**a**) Co 2*p*, (**b**,**c**) Fe 2*p*, (**d**–**f**) Ni 2*p* and (**g**–**i**) O 1*s* core levels of (**a**,**d**,**g**) Ni85Co15_400, (**b**,**e**,**h**) Ni85Fe15_400, and (**c**,**f**,**i**) Ni85Fe15_800 oxides.

The species present on the electrocatalyst surface were identified by XPS. XPS survey spectra (Figure S18) confirm the presence of nickel, oxygen, and adventitious carbon in all electrocatalysts. Cobalt is further present on the surface of Ni85Co15_400 oxide, while iron is detected on the surface of Ni85Fe15_400 and Ni85Fe15_800. High-resolution X-ray photoelectron (HRXPS) spectra of the core levels in bimetallic and reference monometallic oxides are displayed in Figure 5 and Figure S19, respectively. They provide qualitative and quantitative insights into the oxidation states of surface species in the electrocatalysts.

In Ni85Co15_400, the strong satellite peaks appearing in the spectrum of Co 2*p* core level (Figure 5a) at ca. 786 and 803 eV suggest that cobalt is mainly present as Co(II) [72]. Two different divalent cobalt species, in a 37:63 ratio, contribute to the Co 2*p_3/2_* region, namely, Co(II) in CoO environment (at ca. 779.6 eV), and Co(II) in Co(OH)_2_ environment (at 781.3 eV) [73,74]. Recent studies on OER electrocatalysts have reported that the presence of hydroxide species on the defective catalyst surface is beneficial for the surface restructuring process during activation [74]. In Ni85Fe15_400 and Ni85Fe15_800, two different iron species contribute to the main 2*p_3/2_* spin-orbit component (Figure 5b,c), namely, Fe(II) in FeO environment (at ca. 711.3 eV) and Fe(III) in Fe_2_O_3_ environment (at 713.6 eV) [75,76], in agreement with the results of XRD and MRS analyses that indicate the co-presence of RS and SP phases. The relative amount of Fe(II) species decreases from 44% in Ni85Fe15_400 to 37% Ni85Fe15_800, while the relative amount of Fe(III) species oppositely increases from 56% to 63%, as expected due to the increase in the calcination temperature [41]. Regardless of the details, in both samples, Fe(III) species are more abundant than Fe(II) ones. Furthermore, the lack of any obvious contribution from metallic iron, in spite of the formation of a CM phase revealed by Rietveld refinements to the XRD data, is in line with other literature reports on the presence of more oxidized species on the surface of the oxides [41].

Indeed, the HRXPS spectrum in the region of Ni 2*p* core level (Figure 5d) closely resembles that of defective NiO [77]. This finding also agrees with the detection of a pure RS phase in Ni85Co15_400. The main Ni 2*p_3/2_* spin-orbit component clearly reveals two distinct features at approximately 853.7 and 855.5 eV. They correspond to Ni(II) in NiO environment (32%) and Ni(OH)_2_ environment (68%), respectively [74,77,78]. In monometallic Ni100 oxide (Figure S19a), the two peaks are located at slightly higher binding energies (BEs, 854.0 and 855.9 eV) and are in comparable areal ratio (34:66). A similar situation is observed in In Ni85Fe15_800 (Figure 5f) with 854.2 and 855.9 eV peaks in 29:71 areal ratio. In Ni85Fe15_400 (Figure 5e), the areal ratio inverts (66:34) and the peaks shift further towards higher BEs (856.0 and 857.9 eV), as in the case of nickel ferrite [79], again in agreement with the results of XRD and MRS analyses.

In reference to NiH100 oxide (Figure S19b), the 2*p*_3/2_ and 2*p*_1/2_ spin-orbit components (855.4 and 873.0 eV, respectively) exhibit the spin-energy separation (17.6 eV) peculiar to Ni(OH)_2_ [80], with the shake-up satellites centered at 861.6 and 879.6 eV. In this sample, the HRXPS spectrum in the region of O 1*s* core level (Figure S19d) consists of two features that can be ascribed to the Ni-OH bond (530.6 eV) and adsorbed water (531.6 eV) [78,80].

The O 1*s* HRXPS spectra of the other monometallic reference oxide (Figure S19c) and of Ni85Co15_400 (Figure 5g) display three distinct features at 529.5, 531.3, and 532.2 eV. They can be attributed to lattice oxygen (O_L_), surface oxygen vacancies (O_V_), where surface oxygen anions adjacent to lattice vacancies are passivated with hydrogen [41,74,81], and adsorbed O species (O_A_), respectively [41,74]. The concentration of surface O_V_s, which can be beneficial to the OER as they act as OH^−^ adsorption sites [82,83], is slightly higher in Ni85Co15_400 (39.6%) and Ni85Fe15_800 (38.8%, Figure 5i) than in Ni100 (35.0%). Finally, a weak additional contribution, ascribable to adsorbed or chemisorbed O-species such as O_2_ or H_2_O [41,74], is detected at higher BEs in the O 1*s* HRXPS spectrum of Ni85Fe15_400 (Figure 5h). The very strong intensity of the peak at 531.5 eV reveals that the surface of this sample is highly defective, with 62% O_V_s.

#### 3.2.5. Species on the Surface of HER Electrocatalysts

Figure S20 displays the XPS survey spectra of the reduced oxides evaluated as HER electrocatalysts. Nickel, oxygen, and adventitious carbon are still detected in all electrocatalysts, but compared to the adventitious carbon peak, the intensity of the oxygen peak becomes weaker (compare Figures S18 and S20), which confirms the successful reduction of the oxide surface.

**Figure 6:**
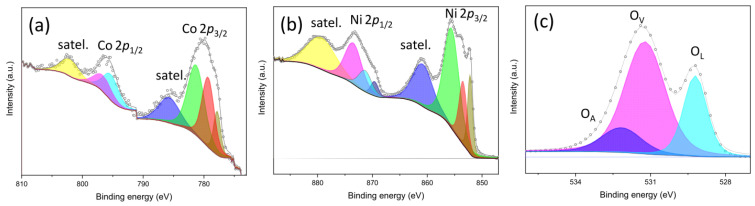
High-resolution XPS curves and fitting in the regions of (**a**) Co 2*p*, (**b**) Ni 2*p*, and (**c**) O 1*s* core levels of Ni85Co15_400R reduced oxide.

Figure 6 displays HRXPS spectra of Co 2*p*, Ni 2*p*, and O 1*s* core levels in Ni85Co15_400R reduced oxide. The intensity of satellite peaks in the spectrum of Co 2*p* core level (Figure 6a) weakens, and a new peak at ca. 777.8 eV appears alongside those at ca. 786 and 803 eV, proving the formation of metallic Co species on the sample surface [84]. Analogously, in the spectrum of Ni 2*p* core level (Figure 6b), the detection of an additional contribution at ca. 852.2 eV confirms the formation of metallic Ni species [85], in full agreement with the indications provided by Rietveld refinements to the XRD data. Moreover, in the O 1*s* HRXPS spectrum (Figure 6c), the intensity of O_L_ peak dramatically reduces (25.5%), while that of O_V_ remarkably intensifies (61.0%), so as their intensity ratio changes from 1.45 to 0.42, confirming the occurrence of partial oxide reduction.

After reduction, the contribution of metallic Ni species is detected in all remaining Ni-containing samples examined (Ni100R, NiH100R, Ni85Fe15_400R, and Ni85Fe15_800R), even if with different intensities. In the O 1*s* spectra of samples Ni100R (Figure S21f) and Ni85Fe15_800R (Figure S21t), a significant weakening of the lowest BE peaks signals the diminishing of O_L_-species concentration, again confirming the partial reduction of the sample surface. No similar change is observed in sample Ni85Fe15_400R (Figure S21r), whose surface is probably reduced to a little extent, in agreement with the indications that emerged from Rietveld refinements to XRD data (Table 3). No obvious change is observed in the Fe 2*p* spectra (Figure S21m–p), indicating that iron is hardly reduced. Finally, in the O 1*s* spectrum of sample NiH100R (Figure S21h), the peak undergoes a decrease in intensity and a shift towards lower BEs.

### 3.3. Electrochemical Performance of the Catalysts

As mentioned above, electrochemical tests were performed on five selected pairs of samples. Their choice was based on the search for the best compromise between high phase purity and small average crystal size. Table 4 reports the codes of MEAs fabricated using them.

After assembling the MEAs in a single cell, a short (two hours) conditioning was carried out in galvanostatic mode at low current density (50 mA cm^−2^) and 50 °C in order to promote their activation (Figure S22). After initial variation, the cell potential remained quite stable for all MEAs. Only a slight increase over time was observed for the MEAs (MEA 1 and MEA 2) based on reference monometallic oxides and for (Ni85Fe15_800-based) MEA 5; on the contrary, the cell potential of (Ni85Co15_400-based) MEA 3 and (Ni85Fe15_400-based) MEA 4 slightly decreased. At the end of the conditioning, (Ni100-based) MEA 2 and (Ni85Fe15_800-based) MEA 5 achieved the highest potential (1.84 V), whereas (Ni85Co15_400-based) MEA 3 and (Ni85Fe15_400-based) MEA 4 exhibited lower voltage (1.78 V). The best performance was observed for the MEA based on materials produced by the CP method (NiH100-based) MEA 1 that reached the lowest cell potential (1.74 V) operating at the same current density.

**Figure 7:**
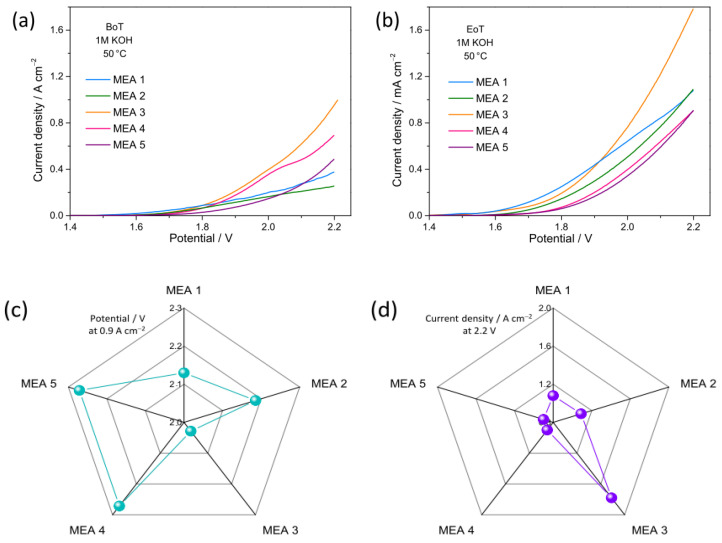
(**a**,**b**) AEMWE cell polarization curves. The shown data refer to the (**a**) beginning of test (BoT) and (**b**) end of test (EoT); for an easier comparison, the same scales are used for both plots. (**c**) Cell potential at 0.9 A cm^−2^ and (**d**) current density at 2.2 V.

Figure 7a,b compares the polarization curves of the cells investigated at the beginning (BoT, Figure 7a) and end of tests (EoT, Figure 7b). The performance of the present MEAs compares well with those reported in the literature for AEMWE cells (Table S4). At the EoT, all MEAs show an improvement (i.e., higher current density at lower potential) with respect to the BoT. The highest current density at the cut-off voltage (2.2 V) is always achieved by MEA 3: 1 A cm^−2^ at the BoT and 1.78 A cm^−2^ at the EoT. At 50 °C, there are no significant differences in activation losses. The MEA performance at the EoT is ranked (Table 4) by comparing the values of cell potential at a given current density (0.9 A cm^−2^, i.e., the maximum value reached by MEA 4 and MEA 5 at the cut-off voltage) and those of current density at a given cell potential (2.2 V). The lower the cell voltage for fixed current density (Figure 7c) and the higher the current for fixed cell voltage (Figure 7d), the better the cell electrochemical performance. Based on these criteria, the performance improves in the order MEA 4 = MEA 5 < MEA 2 < MEA 1 < MEA 3.

Although further investigations would be necessary, the above results confirm that, regardless of the synthesis method, the phase purity (Figure S17a,b) of the anode material has a great influence on the MEA performance. Higher current densities and/or lower potentials are generally obtained with highly pure OER electrocatalysts, in agreement with recent literature reports [71]. Instead, the phase purity of the cathode material seems to be less critical than that of the anodic one, in line with the widely agreed assessment that OER represents the bottleneck of the WE process. In fact, among MEAs with a single-phase anode material (MEA 1-3), the highest current density (~1.8 A cm^−2^) pertains to MEA 3, whose cathode material has only 93% purity rather than to those with 100% purity (Figure S17).

Also, the synergy between metals seems to contribute to enhancing the electrochemical performance, both in terms of HER and OER [86]. Actually, in the present case, MEA 3 outperforms the two MEAs based on the monometallic reference electrocatalysts. However, the impact of the synergy appears to be lower than that of phase-purity, since MEA 4 and MEA 5, pairing low-purity anode (77–81%) and cathode (72–91%) materials, are able to deliver relatively low current density (<1 A cm^−2^) despite their bimetallic nature.

The crucial role played by reactive surface defects in boosting the OER has been frequently highlighted in the literature. Recent studies have pointed out that both surface hydroxide species [74] and O-vacancies play a beneficial role towards OER [41], while smaller-sized crystals generally allow shorter charge migration paths on their surface [41]. Indeed, large amounts of Co(OH)_2_ and Ni(OH)_2_ are detected by XPS on the highly defective surface of the finely grained Ni85Co15_400, together with a high concentration of O_v_s (39.6%). In Ni85Fe15_800, on whose surface a comparable O_v_-amount (38.8%) is present, oxide crystals are larger (55 against 42 nm), and this may hamper the charge migration. As O_v_s behave as a descriptor for the OER process [41], an optimal range of concentration exists. This explains why the huge Ov-amount (62%) in Ni85Fe15_400 ultimately proves detrimental, and the electrochemical performance of MEA 4 is unsatisfactory, despite the relatively small crystal size (28 nm) could be beneficial for charge migration.

**Figure 8:**
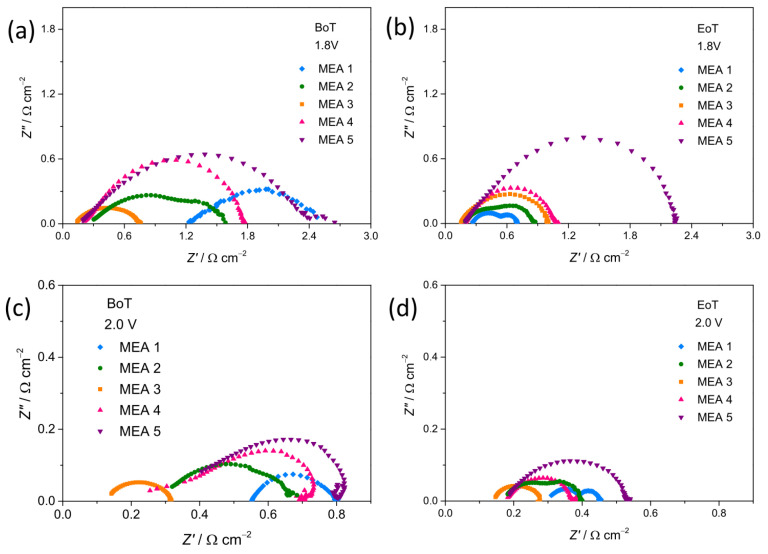

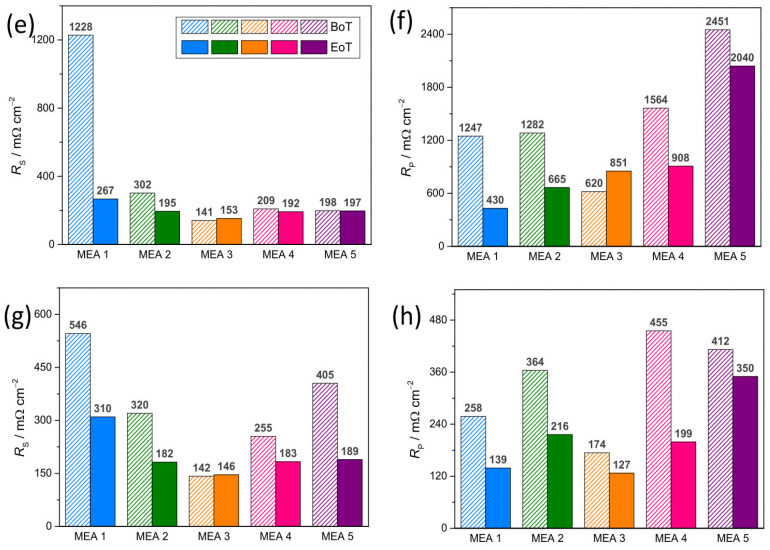
(**a**–**d**) Nyquist plots at different potentials: (**a**,**b**) 1.8 V and (**c**,**d**) 2.0 V. The shown data refer to the (**a**,**c**) beginning of test (BoT) and (**b**,**d**) end of test (EoT). At each potential, the same scales are used for an easier comparison. (**e**,**g**) *R*_S_ and (**f**,**h**) *R*_P_ values at (**e**,**f**) 1.8 V and (**g**,**h**) 2.0 V at BoT and EoT.

Figure 8a–d compares EIS spectra recorded on all MEAs at 50 °C at 1.8 and 2.0 V cell potential. At higher potential (2 V), clear evidence of at least two distinct semicircles appears in the Nyquist plots. The semicircle with low frequency, attributed to the cathode, is significantly larger than one with high frequency, which is associated with the anode. After the durability test (Figure 8d), the semicircles appear significantly overlapped. It is possible that the anode semicircle shifts towards lower frequencies due to reversible losses at the anode.

In order to more deeply understand the electrochemical performance described above, the series resistance (*R*_S_) and polarization resistance (*R*_P_) were estimated from the Nyquist plots, as reported in Section 2.5. The MEA activation during operation, a process involving both catalysts and membrane, can be responsible for the observed enhancement in cell performance. Improved interfacial contact between the membrane and the electrode likely contributes as well. The significant decrease in the values of both *R*_S_ and *R*_P_ supports these hypotheses for most of the MEAs.

Regardless of the cell potential (1.8 or 2.0 V), MEA 3 shows lower *R*_S_ than other MEAs (Figure 8e,g), both before and after the tests. Therefore, MEA 3 is the most efficient. This behavior confirms that the MEA performance is greatly influenced by the phase purity and average crystal size of the electrode materials. Although further investigations would be necessary, the synthesis method seems to affect the performance: Under the same conditions, all MEAs prepared using materials synthesized via the SG method show lower *R*_S_ than MEA based on materials produced by the CP method (NiH100-based MEA 1).

The *R*_P_ values displayed in Figure 8f,h reveal that MEA 3 generally exhibits a better interface with the membrane. Forming a proper interface with the membrane decreases the mass transfer constraints due to the accumulation of developed O_2_ in the catalytic layer.

**Figure 9:**
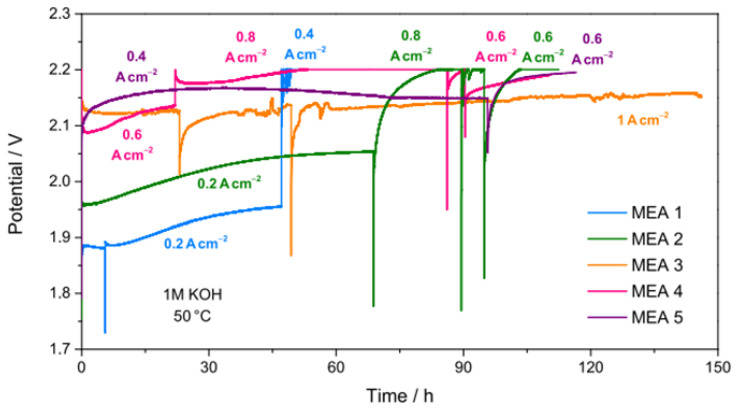
Durability tests at 50 °C and ambient pressure for all MEAs.

Figure 9 displays the variation undergone by the cell voltage during the durability tests, carried out at 50 °C by recirculating a 1 M KOH solution to the anode side. The values of current densities, cell voltages, and durability times can be found in Table S5. Only MEA 3 is able to reach a current density of 1 A cm^−2^. The remaining ones operate at lower current densities, with their performance progressively degrading over time until reaching the set cut-off voltage (2.2 V). Reversible losses are observed during start-up and stop cycles. A possible explanation could be the occurrence of some diffusion limitations that influence the release of the product gas. Due to reversible losses during cycles, all durability test curves present a slightly lower voltage efficiency than steady-state operation.

By comparing the values of current densities, cell voltages and durability times reported in Table S5, it emerges that the only MEA prepared using materials produced by the CP method (MEA 1) reaches the cut-off voltage (2.2 V) at low current density (0.4 A cm^−2^) after solely 48 h of durability test. For MEA 2, MEA 4, and MEA 5, durability time is longer (120 h), but they never reach a current density higher than 0.8 A cm^−2^. The best performance (1 A cm^−2^ at 2.15 V), during a steady-state 150 h-long stability test with 1 M KOH recirculating through the cell, is achieved with the MEA 3 that outperforms the remaining ones also in this test. Hence, Ni85Co15_400-based MEA is the most successful in a zero-gap AEMWE full cell.

### 3.4. Structural Stability of the Electrocatalysts

The structural stability of all electrocatalysts was evaluated by carrying out XRD analysis on the fresh and reacted electrodes (Figure S23). The signals arising from the electrode supports are clearly observed both before and after the reaction. In addition to the XRD peaks from the Ni-felt, the reflections from the crystallographic planes of the reference β-Ni(OH)_2_ and NiO electrocatalysts are detected in both the fresh and reacted anodes of MEA 1 (Figure S23a) and MEA 2 (Figure S23c), respectively. Analogously, the reflections from the crystallographic planes of the RS-structured single/main component of the catalysts in their oxidized form, clearly visible in the fresh anodes of MEA 3 (Figure S23e) and MEA 4 (Figure S23g), are still detected after reaction. These findings prove the structural integrity of the anode materials: Although, obviously, their surface is modified during the reaction, the bulk structure of the pristine material remains unchanged [87]. Similar considerations apply to cathode materials. In addition to the signal from the carbon paper [88], the diffraction peaks from the CM (single/main component of the catalysts in their reduced form) are detected in both the fresh and reacted cathodes of all MEAs (Figure S23b,d,f,h).

## 4. Conclusions

A very simple scalable method for the synthesis of efficient and low-cost electrocatalysts for alkaline water electrolysis is proposed. Nanostructured NiCo- and NiFe-oxides are prepared by the sol–gel method and subsequent calcination in air at different temperatures and then evaluated as anode materials in a zero-gap AEMWE full cell. The cell cathodes are fabricated using the same materials after reduction in a H_2_/Ar atmosphere.

The results of electrochemical tests clearly point to a significant role played by the nanomaterial phase purity and average crystal size in determining cell performance. Higher current densities at lower overpotentials are obtained using highly pure and finely grained electrocatalysts. The comparison with reference MEAs based on monometallic oxides suggests that the synergistic action between metals can contribute to enhancing the electrochemical performance.

Among those investigated, MEA joining Ni85Co15_400 anode (consisting of ~36 nm-sized 100% purity rock-salt oxide crystals with abundant O_v_s and hydroxide species on their highly defective surface) and Ni85Co15_400R cathode (consisting of ~56 nm-sized 93% purity cubic metal/alloy) outperforms the remaining ones. It exhibits a current density of 1 A cm^−2^ at 2.15 V during a steady-state 150 h long stability test with 1 M KOH recirculating through the cell, the lowest *R*_S_ at any cell potential (1.8 or 2.0 V), and the highest current density at the cut-off voltage (2.2 V) both at the BoT (1 A cm^−2^) and EoT (1.78 A cm^−2^).

Although there is still room for improvement, the presented results demonstrate, as proof of concept, the possibility of producing green hydrogen via AEMWE using a very simple and scalable process for the synthesis of nanostructured NiCo- and NiFe-based cell electrode materials.

## Figures and Tables

**Table 1:** Codes, composition, and calcination temperature (*T*_C_) of the nanostructured oxides prepared via the sol–gel (SG) method.

Code	Metal Molar Concentrations			*T*_C_/°C
	Ni	Co	Fe	
Ni100	1.00			400
Ni85Co15_400	0.85	0.15		400
Ni85Co15_800	0.85	0.15		800
Ni50Co50_400	0.50	0.50		400
Ni50Co50_800	0.50	0.50		800
Ni85Fe15_400	0.85		0.15	400
Ni85Fe15_800	0.85		0.15	800

**Table 2:** Main results of Rietveld analysis on the OER electrocatalysts. The relative abundance of each phase is reported, together with the mean crystallite size (*d*). In the case of a multiphase material, the *d*-value is calculated as a weighted average over the phases present. RS, CM, and SP stand for rock-salt, cubic metal/alloy, and spinel structure, respectively.

Code	Phase(s) Abundance/wt%				*d*/nm
	β-Ni(OH)2	RS	CM	SP	
NiH100	100.0				24
Ni100		100.0			21
Ni85Co15_400		100.0			36
Ni85Co15_800		100.0			105
Ni50Co50_400		46.8	30.6	22.6	42
Ni50Co50_800		72.2		27.8	105
Ni85Fe15_400		77.2	12.4	10.4	28
Ni85Fe15_800		81.4		18.6	55

**Table 3:** Main results of Rietveld analysis on the HER electrocatalysts. The relative abundance of each phase is reported, together with the mean crystallite size (*d*). In the case of multiphase material, the *d*-value is calculated as a weighted average over the phases present. RS, CM, and SP stand for rock-salt, cubic metal/alloy, and spinel structure, respectively.

Sample	Phase(s) Abundance/wt%			*d*/nm
	CM	RS	SP	
NiH100R	100.0			61.3
Ni100R	100.0			61.0
Ni85Co15_400R	93.3	6.7		55.9
Ni85Co15_800R	100.0			139.4
Ni50Co50_400R	81.8	18.2		39.4
Ni50Co50_800R	100.0			88.6
Ni85Fe15_400R	71.7	18.5	9.8	38.5
Ni85Fe15_800R	91.1	2.9	6.0	59.5

**Table 4:** Codes and composition of the membrane-electrode assemblies (MEAs) electrochemically tested. Percentage of ionomer: 20%, for both electrodes. Ionomer: Fumatech^®^ ION FAA3 (10%). Membrane: Fumatech^®^FAA3-50. Value of cell potential (*V*_C_) and current density (*J*) achieved by all MEAs at fixed *J* (0.9 A cm^−2^) and *V*_C_ (2.2 V), respectively.

MEA Code and Composition		Cathode	Anode	*V*_C/_V	*J*/A cm^−2^
1	NiH100-based	NiH100R	NiH100	2.13	1.08
2	Ni100-based	Ni100R	Ni100	2.14	1.09
3	Ni85Co15_400-based	Ni85Co15_400R	Ni85Co15_400	2.03	1.78
4	Ni85Fe15_400-based	Ni85Fe15_400R	Ni85Fe15_400	2.20	0.90
5	Ni85Fe15_800-based	Ni85Fe15_800R	Ni85Fe15_800	2.20	0.90

## Data Availability

Data are contained within the article or Supplementary Materials.

## Supplementary Materials

The following supporting information can be downloaded at https://www.mdpi.com/article/10.3390/nano15131042/s1, Figure S1: Schematic description of the experimental procedure followed to prepare nanostructured oxides to be used as OER electrocatalysts at the cell anode; Figure S2: Synthesis scheme of anode electrocatalyst via co-precipitation method; Figure S3: Schematic description of the experimental procedure for the preparation of nanostructured HER electrocatalysts (cathode materials); Table S1: Codes and reduction temperatures (*T*_R_) of each oxide; Figure S4: TPR profiles of the bimetallic oxides in H_2_/Ar atmosphere for 30 min; Figure S5: Airbrush system for manual electrodeposition of catalytic inks; Figure S6: Cathode, anode, Fumatech^®^ membrane, and MEA with 5 cm^2^ active area; Figure S7: Scheme of a single cell unit used for the electrochemical assessment; Figure S8: SEM micrographs and SEM/EDX spectra of OER electrocatalysts; Figure S9: XRD patterns of reference electrocatalysts; Figure S10: Rietveld refinements from XRD data of OER catalysts; Table S2: Results of Rietveld refinements for OER electrocatalysts; Figure S11: Effect of the change in synthesis parameter in terms of phases formed in bimetallic oxides; Figure S12: Micro-Raman spectra, as measured at different random locations, within monometallic Ni100 reference electrocatalyst; Figure S13: Micro-Raman spectra, as measured at different random locations, within each bimetallic oxide; Figure S14: SEM micrographs of HER electrocatalysts; Figure S15: SEM/EDX spectra of HER electrocatalysts; Figure S16: Rietveld refinements for reduced oxides; Table S3: Results of Rietveld refinements for HER electrocatalyst; Figure S17: Phase purity of electrode materials in the (a) oxidized and (b) reduced form and (c) corresponding average crystal size, as resulting from Rietveld refinements to XRD data; Figure S18: Survey XPS spectra of the oxides evaluated as OER electrocatalysts; Figure S19: High-resolution XPS curves and fitting in the regions of Ni 2*p* and O 1*s* core levels of Ni100 and NiH100 samples; Figure S20: Survey XPS spectra of the reduced oxides evaluated as HER electrocatalysts; Figure S21: Comparison between HRXPS spectra of electrocatalysts in the oxidized and reduced form; Figure S22: Conditioning at low current density (50 mA cm^−2^) and 50 °C; Table S4: Performance and durability comparison of different membrane–electrode assemblies in similar AEMWE conditions; Table S5: Current densities, cell voltages, and durability test times for AEM systems fed with 1 M KOH; Figure S23: XRD patterns of anodes and cathodes utilized to fabricate MEA 1, MEA 2, MEA 3, and MEA 4 fresh and after reaction. (References [89,90,91,92] are cited in Supplementary Materials).
