# Supplementary material for: Cost-Effective Bimetallic Catalysts for Green H2 Production in Anion Exchange Membrane Water Electrolyzers
Source: Nanomaterials (Basel). 2025 Jul 4;15(13):1042. doi: 10.3390/nano15131042 (PMC12250770; doi:10.3390/nano15131042)
Supplement: Supplementary file 1 [file nanomaterials-15-01042-s001.zip › nanomaterials-3727185-supplementary.pdf]

Cost-effective bimetallic catalysts for green H_2 production in anion exchange membrane water electrolyzers

S. Campagna Zignani[a], M. Fazio[a], M. Pascale[a], C. Alessandrello[b], C. Triolo[b,c], M. G. Musolino[b,c], S. Santangelo[b,c]

[a] *Institute of Advanced Technologies for Energy (ITAE) of the National Research Council (CNR), 98126 Messina, Italy*
[b] *Department of Civil, Energy, Environmental and Materials Engineering (DICEAM), Mediterranean University of Reggio Calabria, 89122 Reggio Calabria, Italy*
[c] *National Reference Center for Electrochemical Energy Storage (GISEL), National Interuniversity Consortium for the Science and Technology of Materials (INSTM), 50121 Florence, Italy*

Supporting information

Synthesis of OER and HER electrocatalysts

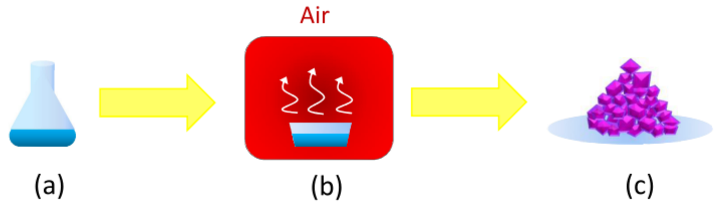

Figure S1. Schematic description of the experimental procedure followed to prepare nanostructured oxides to be used as OER electrocatalysts at the cell anode. (a) Precursor solution (b) gel calcination and (c) as-obtained oxide nanoparticles.

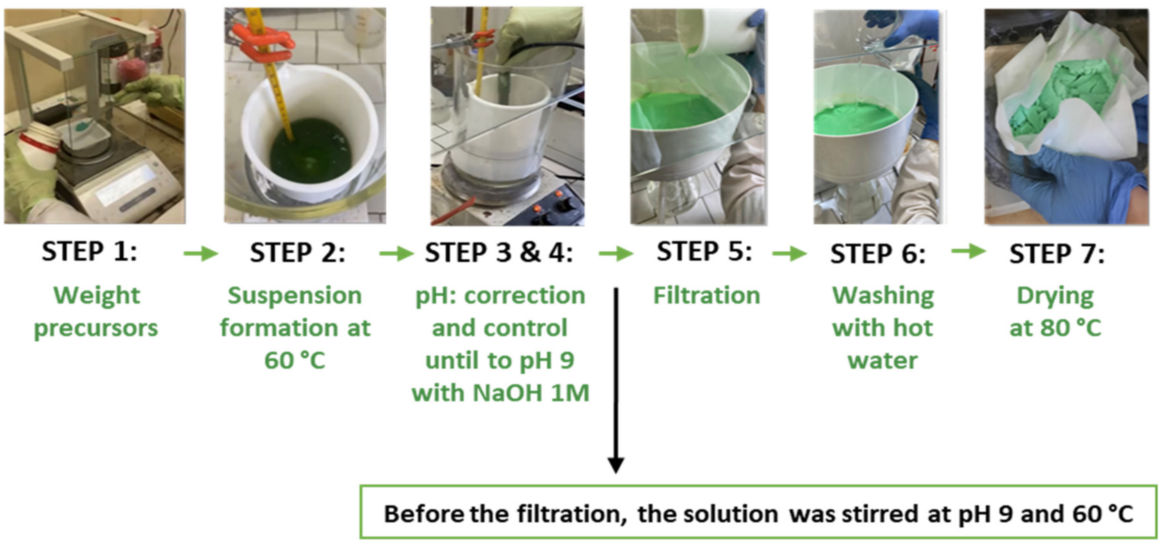

Figure S2. Synthesis scheme of anode electrocatalyst via co-precipitation method.

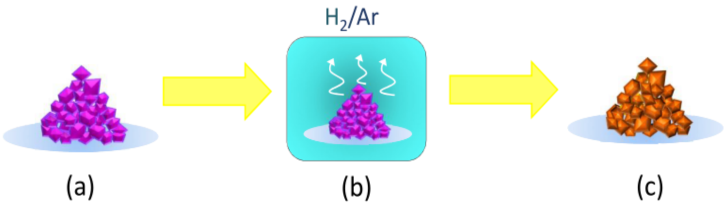

Figure S3. Schematic description of the experimental procedure for the preparation of nanostructured HER electrocatalysts (cathode materials). (a) Pristine oxide (b) reduction in H_2/Ar atmosphere and (c) reduced metallic nanoparticles.

Table S1. Codes and reduction temperatures (T_R) of each oxide, as determined by means the temperature programmed reduction (TPR) analysis.

**Code**	**Pristine oxide code**	**T_R / °C**
NiH100R	NiH100	332
Ni100R	Ni100	467
Ni85Co15_400R	Ni85Co15_400	500
Ni85Co15_800R	Ni85Co15_800	600
Ni50Co50_400R	Ni50Co50_400	362
Ni50Co50_800R	Ni50Co50_800	572
Ni85Fe15_400R	Ni85Fe15_400	467
Ni85Fe15_800R	Ni85Fe15_800	520

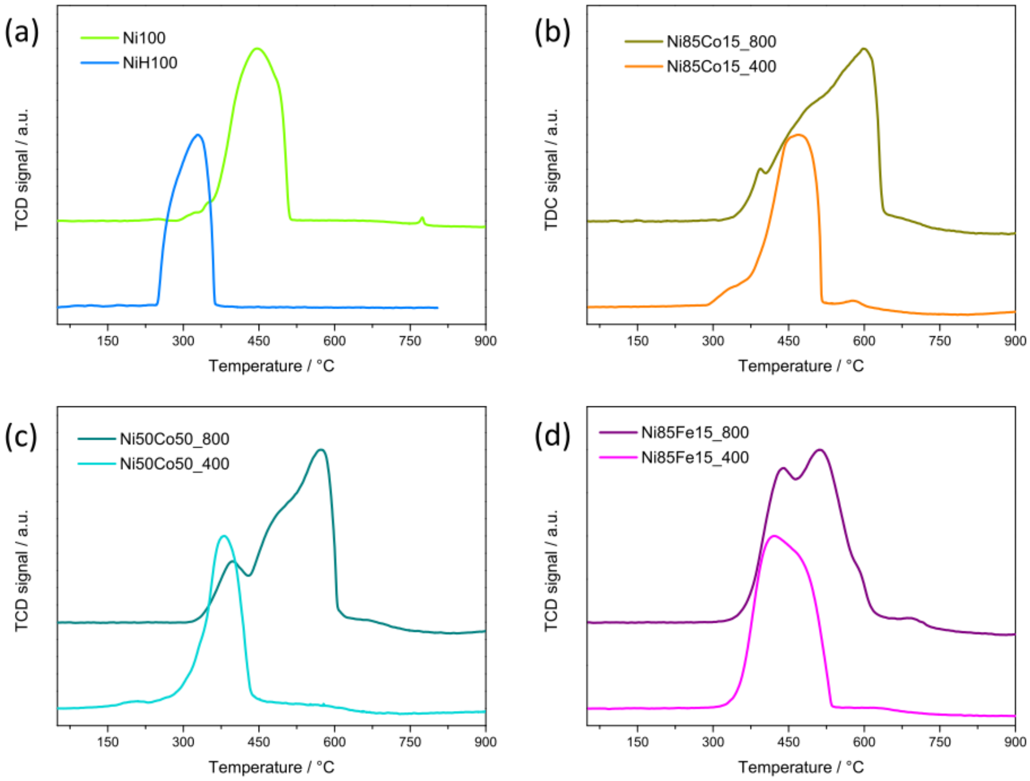

Figure S4. TPR profiles of the bimetallic oxides in H_2/Ar atmosphere for 30 minutes. The shown data refer to (a) reference monometallic catalysts and bimetallic (b) Ni85Co15, (c) Ni50Co50 and (d) Ni85Fe15 oxides.

Fabrication of electrodes and MEAs

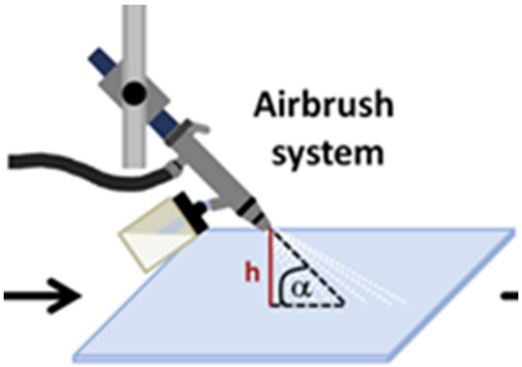

Figure S5. Airbrush system for manual electrodeposition of catalytic inks.

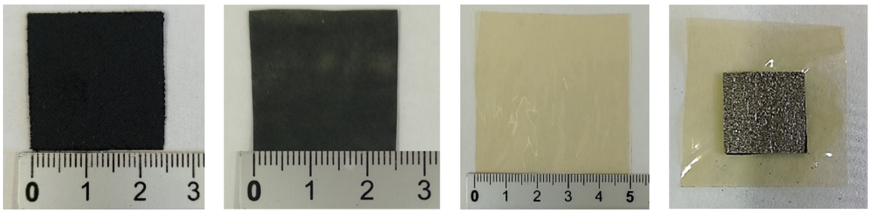

Figure S6. Cathode, anode, Fumatech® membrane and MEA with 5 cm² active area (from left to right).

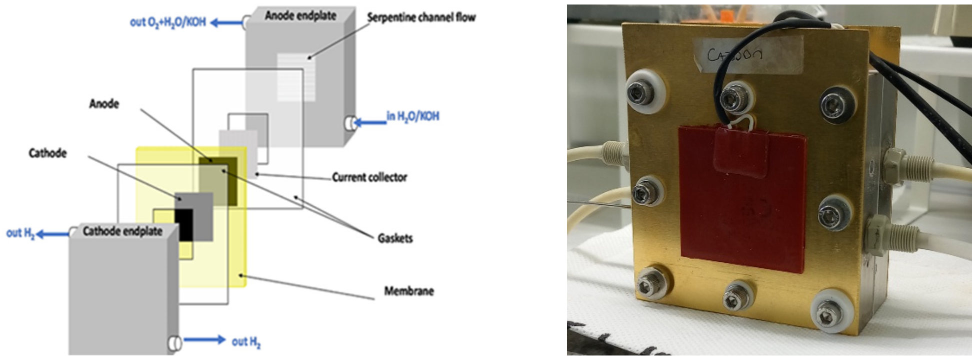

Figure S7. Scheme of a single cell unit used for the electrochemical assessment.

Morphology and composition of OER electrocatalysts

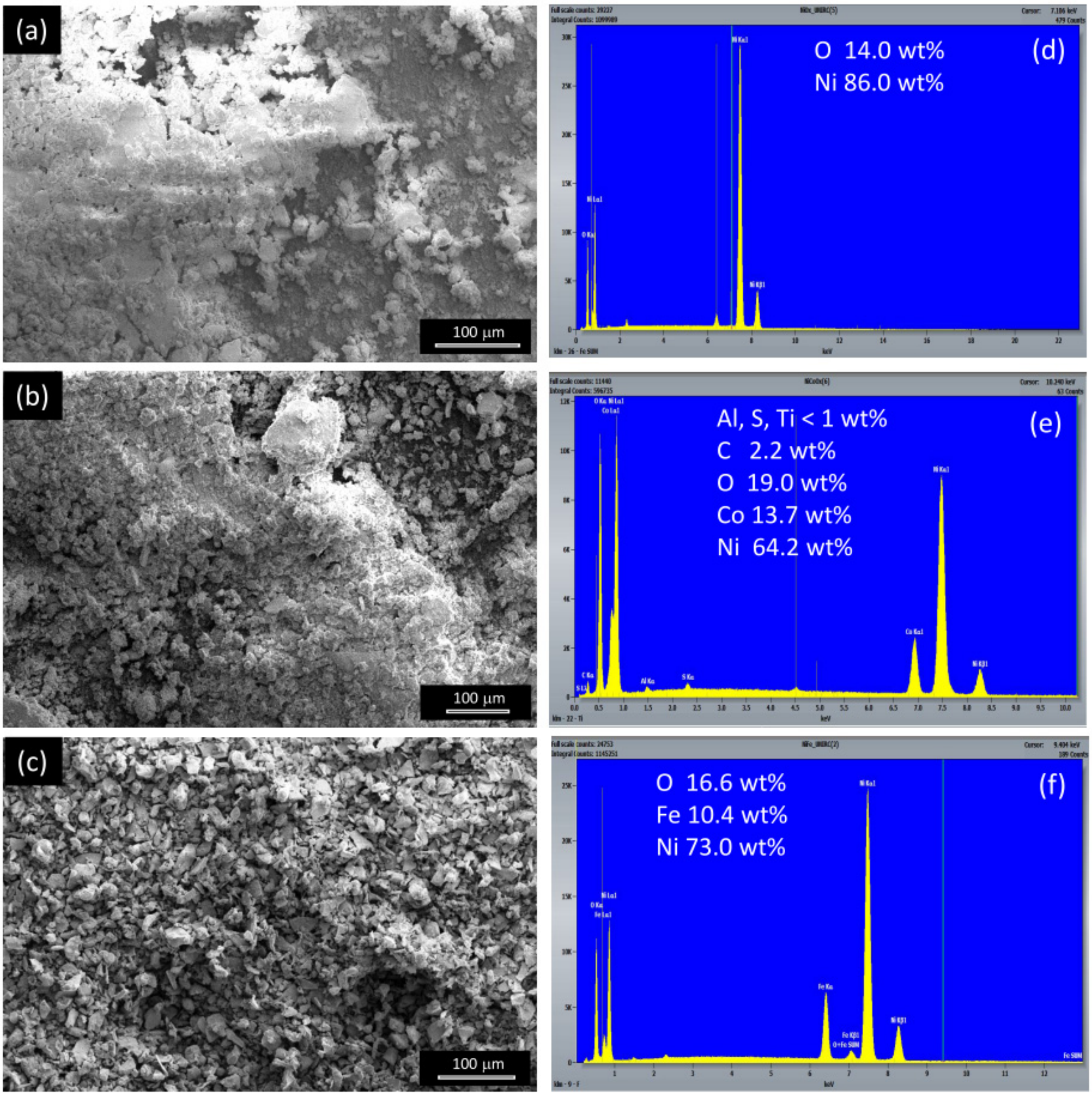

Figure S8. (a–c) SEM micrographs of samples (a) NiH100, (b) Ni85Co15_400 and (c) Ni85Fe15_400. (d–f) SEM/EDX spectra of samples (d) Ni100, (e) Ni85Co15_400 and (f) Ni85Fe15_400. Some environmental contamination is present in sample Ni85Co15_400.

Reference nickel-based OER and HER electrocatalysts

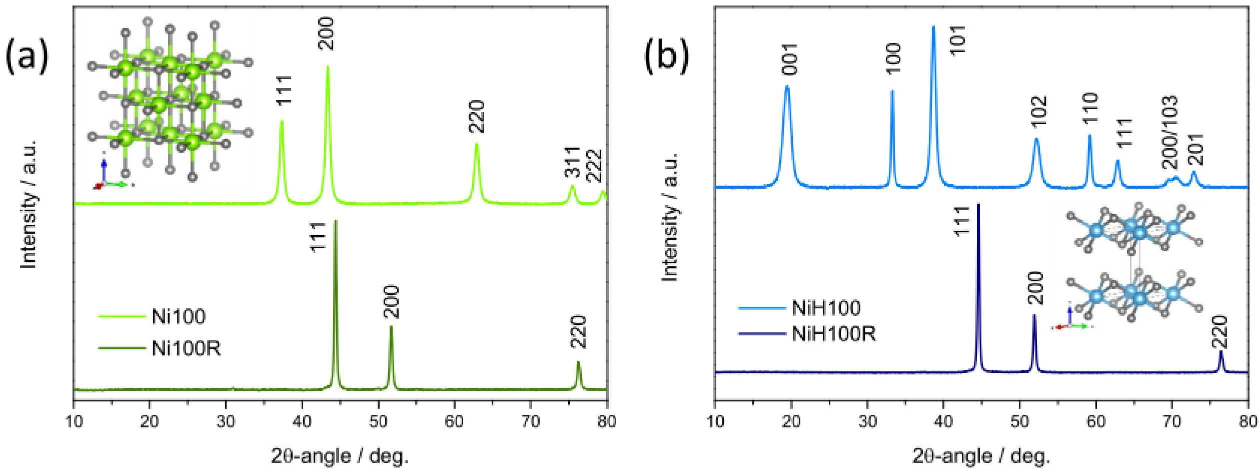

Figure S9. XRD patterns of reference electrocatalysts. The shown data refer to samples (a) Ni100 and Ni100R, and (b) NiH100 and NiH100R.

Rietveld refinements from XRD data of OER catalysts

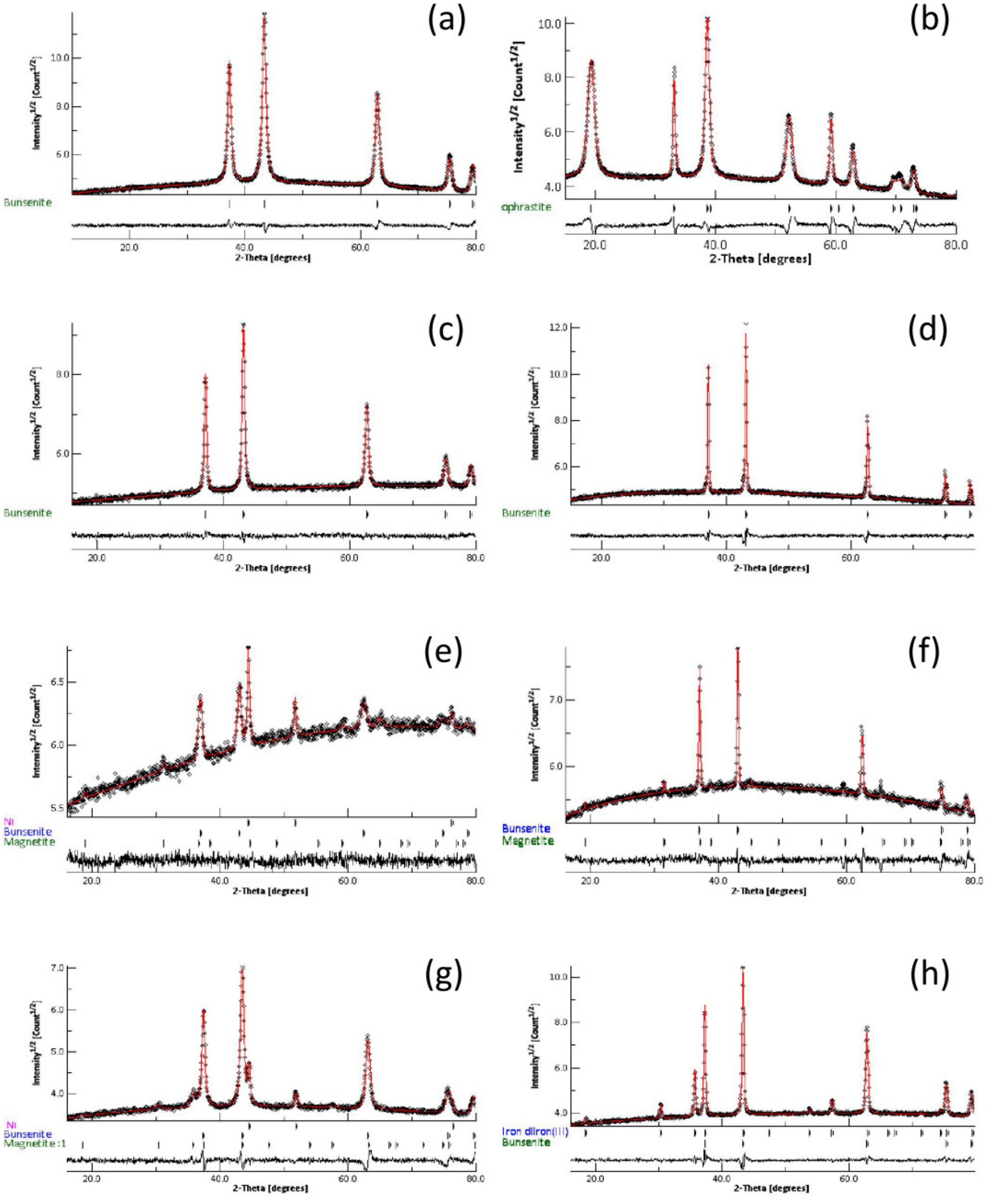

Figure S10. Rietveld refinements for oxides (a) Ni100, (b) NiH100, (c) Ni85Co15_400, (d) Ni85Co15_800, (e) Ni50Co50_400, (f) Ni50Co50_800, (g) Ni85Fe15_400 and (h) Ni85Fe15_800.

Table S2. Results of Rietveld refinements for OER electrocatalysts. Values in parentheses are estimated errors for cell size and microstrain. For multi-phase materials, the mean crystallite size was calculated as the weighted average of these values, where the relative amounts of each phase represent the weights.

Sample	Phase	% w/w	Cell size / Å	Crystal size / nm	Microstrain	*d* / nm
NiH100	β-Ni(OH)$_2$	100.0	*a* = 3.1188(2)<br>*c* = 4.5871(9)	24.3±0.9	NS	24.3
Ni100	RS	100.0	4.17609(6)	21.3 ± 0.1	0.00176(5)	21.3
Ni85Co15_400	RS	100.0	4.18921(6)	35.7 ± 0.4	0.00179(5)	35.7
Ni85Co15_800	RS	100.0	4.1936(1)	105.1 ± 0.1	0.00065(2)	105.1
Ni50Co50_400	RS	46.8	4.2068(6)	44.8 ± 13.7	0.0054(5)	42.0
	CM	30.6	3.5336(2)	37.2 ± 2.1	NS	
	SP	22.6	8.117(4)	42.6 ± 6.0	0.0056(6)	
Ni50Co50_800	RS	72.2	4.2058(1)	107.1 ± 11.0	0.00155(9)	105.3
	SP	27.8	8.037(1)	100.7 ± 21.2	0.00023(13)	
Ni85Fe15_400	RS	77.2	4.1650(2)	25.9 ± 0.5	0.0025(1)	28.3
	CM	12.4	3.5249(4)	33.8 ± 4.0	0.0013(6)	
	Inv SP	10.4	8.317(3)	40.0 ± 4.8	0.0099(8)	
Ni85Fe15_800	RS	81.4	4.1826(2)	68.8 ± 0.2	0.00142(2)	54.6
	SP	18.6	8.3503(6)	51.3 ± 2.1	0.0008(2)	

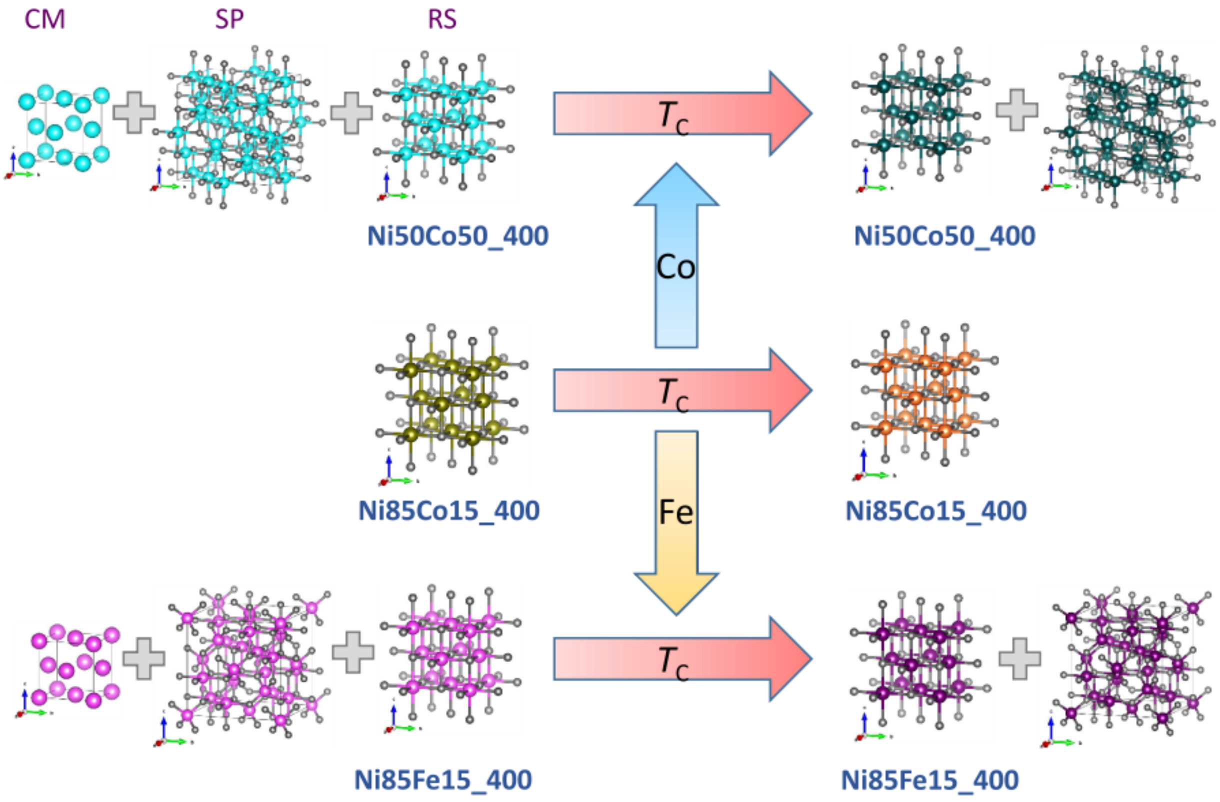

Figure S11. Effect of the change in synthesis parameter in terms of phases formed in bimetallic oxides.

Spatial uniformity of OER electrocatalysts

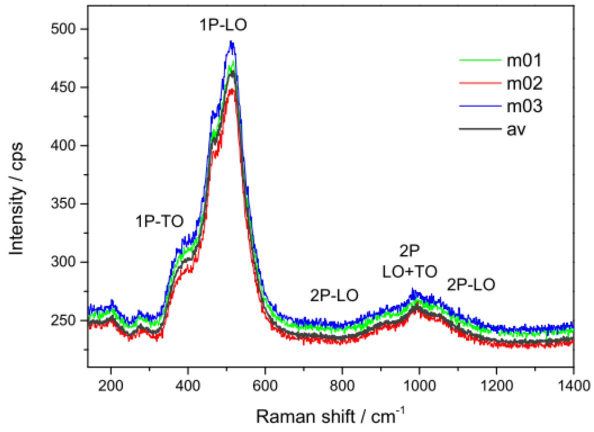

Figure S12. Micro-Raman spectra, as measured at different random locations, within monometallic Ni100 reference electrocatalyst.

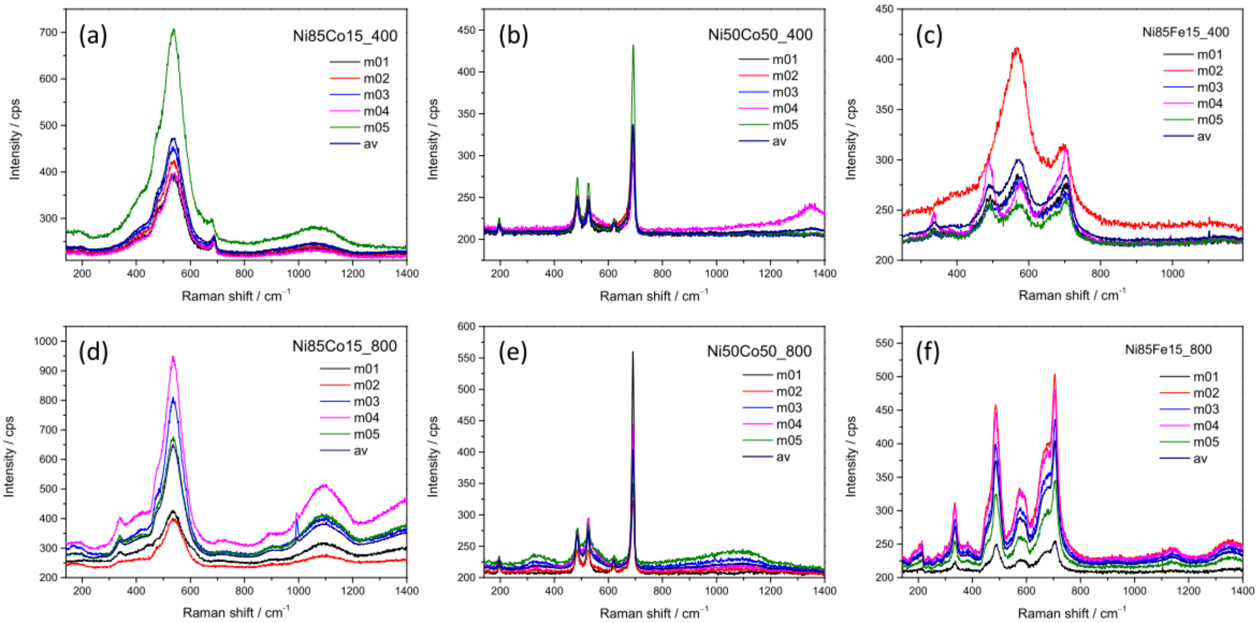

Figure S13. Micro-Raman spectra, as measured at different random locations, within each bimetallic oxide. Spectra refer to samples (a) Ni85Co15_400, (b) Ni50Co50_400, (c) Ni85Fe15_400, (d) Ni85Co15_800, (d) Ni50Co50_800 and (f) Ni85Fe15_800.

Morphology and composition of HER electrocatalysts

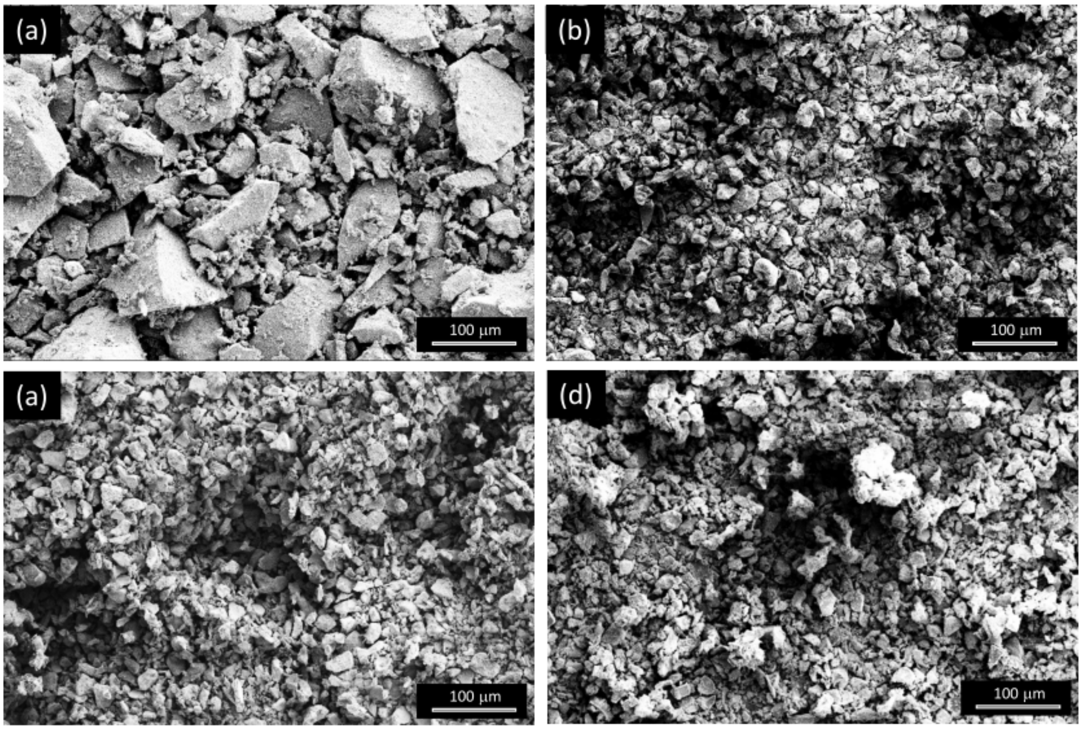

Figure S14. SEM micrographs of samples (a) NiH100R, (b) Ni100R, (c) Ni85Co15_400R and (d) Ni85Fe15_400R.

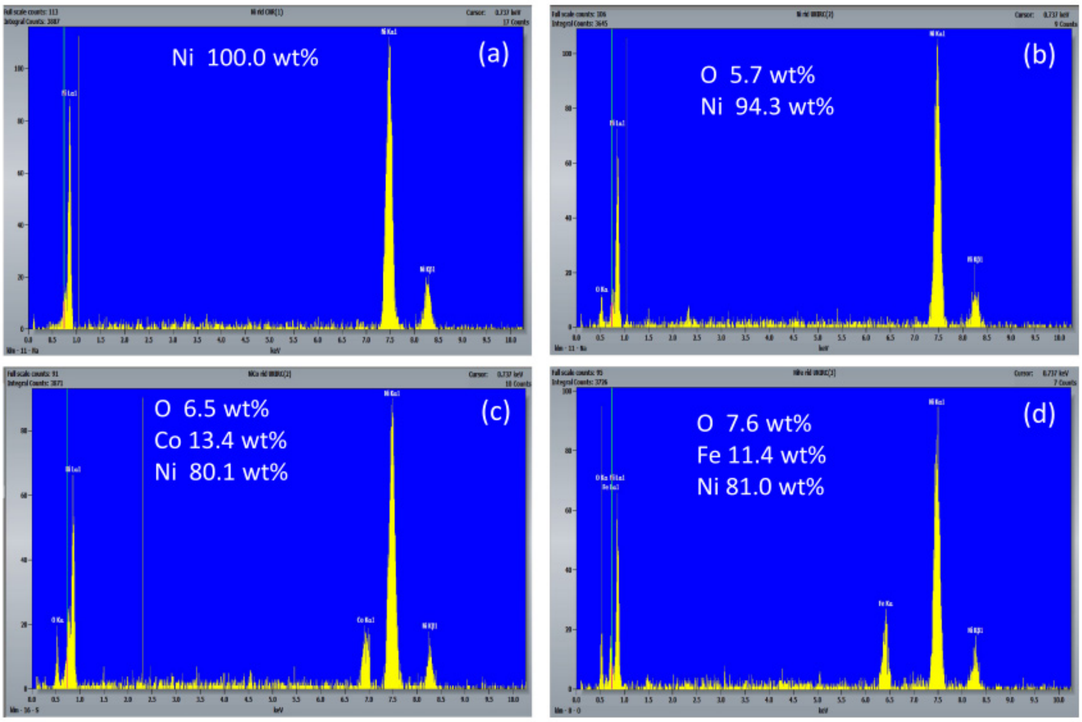

Figure S15. SEM/EDX EDX spectra of samples (a) NiH100R, (b) Ni100R, (c) Ni85Co15_400R and (d) Ni85Fe15_400R.

Rietveld refinements from XRD data of HER catalysts

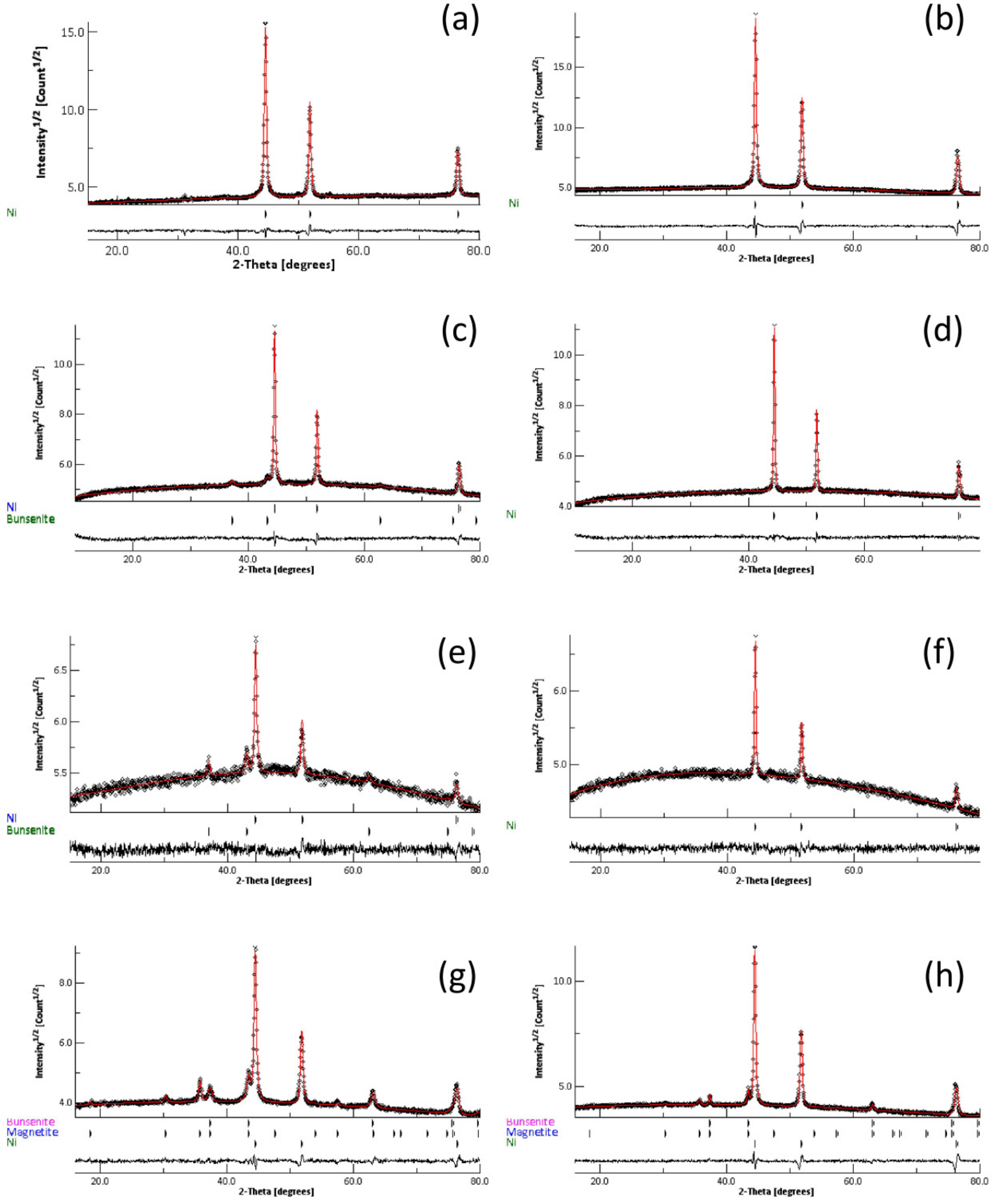

Figure S16. Rietveld refinements for reduced oxides (a) Ni100R, (b) NiH100R, (c) Ni85Co15_400R, (d) Ni85Co15_800R, (e) Ni50Co50_400R, (f) Ni50Co50_800R, (g) Ni85Fe15_400R and (h) Ni85Fe15_800R.

Table S3. Results of Rietveld refinements for HER electrocatalysst. Values in parentheses are estimated errors for cell size and microstrain. For multi-phase materials, the mean crystallite size was calculated as the weighted average of these values, where the relative amounts of each phase represent the weights.

Sample	Phase	% w/w	Cell size / Å	Crystal size / nm	Microstrain	*d* / nm
NiH100R	CM	100.0	3.52044(4)	61.3±4	0.00108(1)	61.3
Ni100R	CM	100.0	3.5253(2)	61.0±0.3	0.00088(2)	61.0
Ni85Co15_400R	CM	93.3	3.52448(4)	56.6±0.7	0.00073(6)	55.9
	RS	6.7	4.184(2)	46.5±6.4	0.0055(7)	
Ni85Co15_800R	CM	100.0	3.5305(2)	139.4±2.2	0.000849(2)	139.4
Ni50Co50_400R	CM	81.8	3.5283(1)	36.7±1.6	0.0013(2)	39.4
	RS	18.2	4.204(1)	51.6±4.3	0.0032(3)	
Ni50Co50_800R	CM	100.0	3.5378(9)	88.6±0.9	0.00160(4)	88.6
Ni85Fe15_400R	CM	71.7	3.52596(7)	40.0±0.7	0.00168(6)	38.5
	RS	18.5	4.170(1)	39.1±6.3	0.0057(2)	
	SP	9.8	8.326(1)	26.0±1.3	NS	
Ni85Fe15_800R	CM	91.1	3.52869(5)	60.6±1.3	0.00164(5)	59.5
	SP	6.0	8.336(2)	42.0±8.5	0.0011(6)	
	RS	2.9	4.168(5)	61.8±14.9	0.004(1)	

Phase purity and crystal size of the catalysts

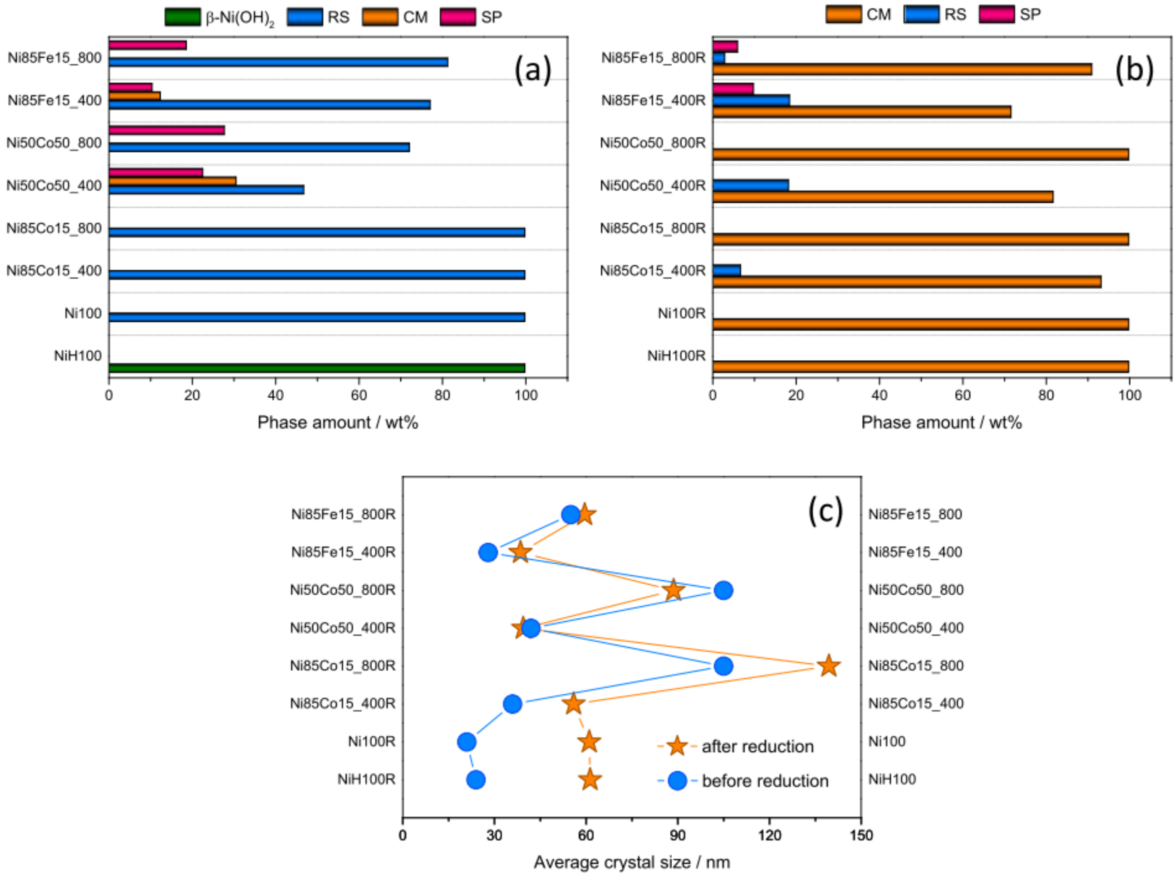

Figure S17. Phase purity of electrode materials in the (a) oxidized and (b) reduced form and (c) corresponding average crystal size, as resulting from Rietveld refinements to XRD data.

Surface composition of the electrocatalysts

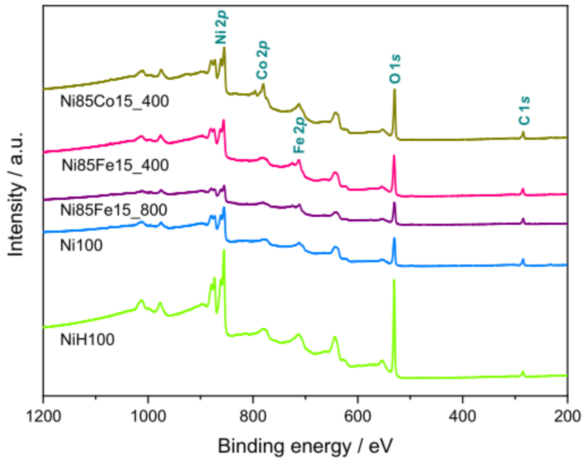

Figure S18. Survey XPS spectra of the oxides evaluated as OER electrocatalysts.

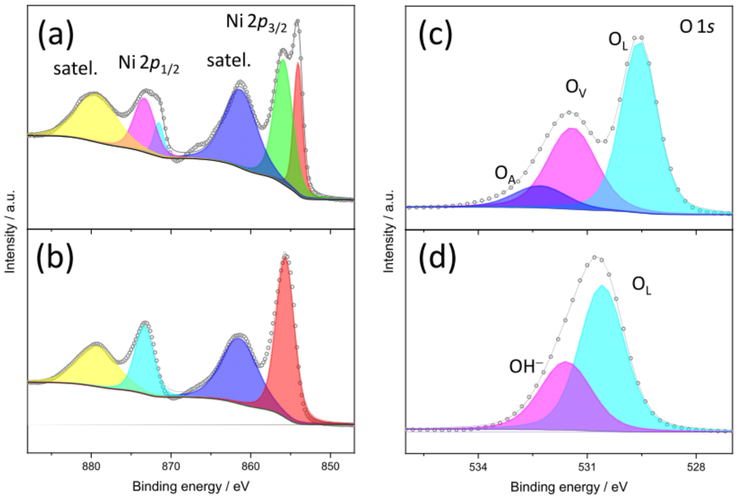

Figure S19. High-resolution XPS curves and fitting in the regions of (a,b) Ni 2*p* and (c,d) O 1*s* core levels of (a,c) Ni100 and (b,d) NiH100 samples.

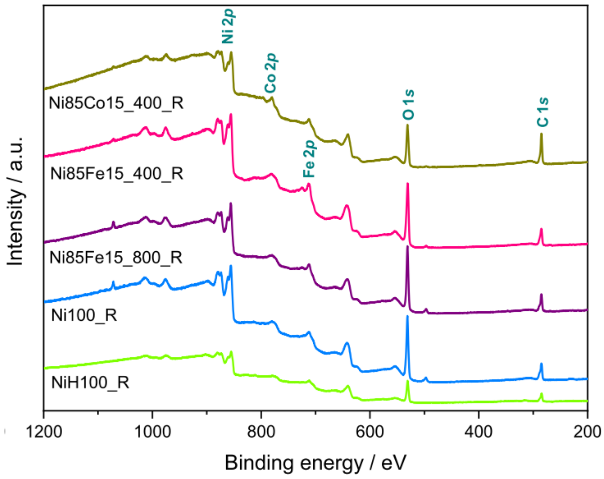

Figure S20. Survey XPS spectra of the reduced oxides evaluated as HER electrocatalysts.

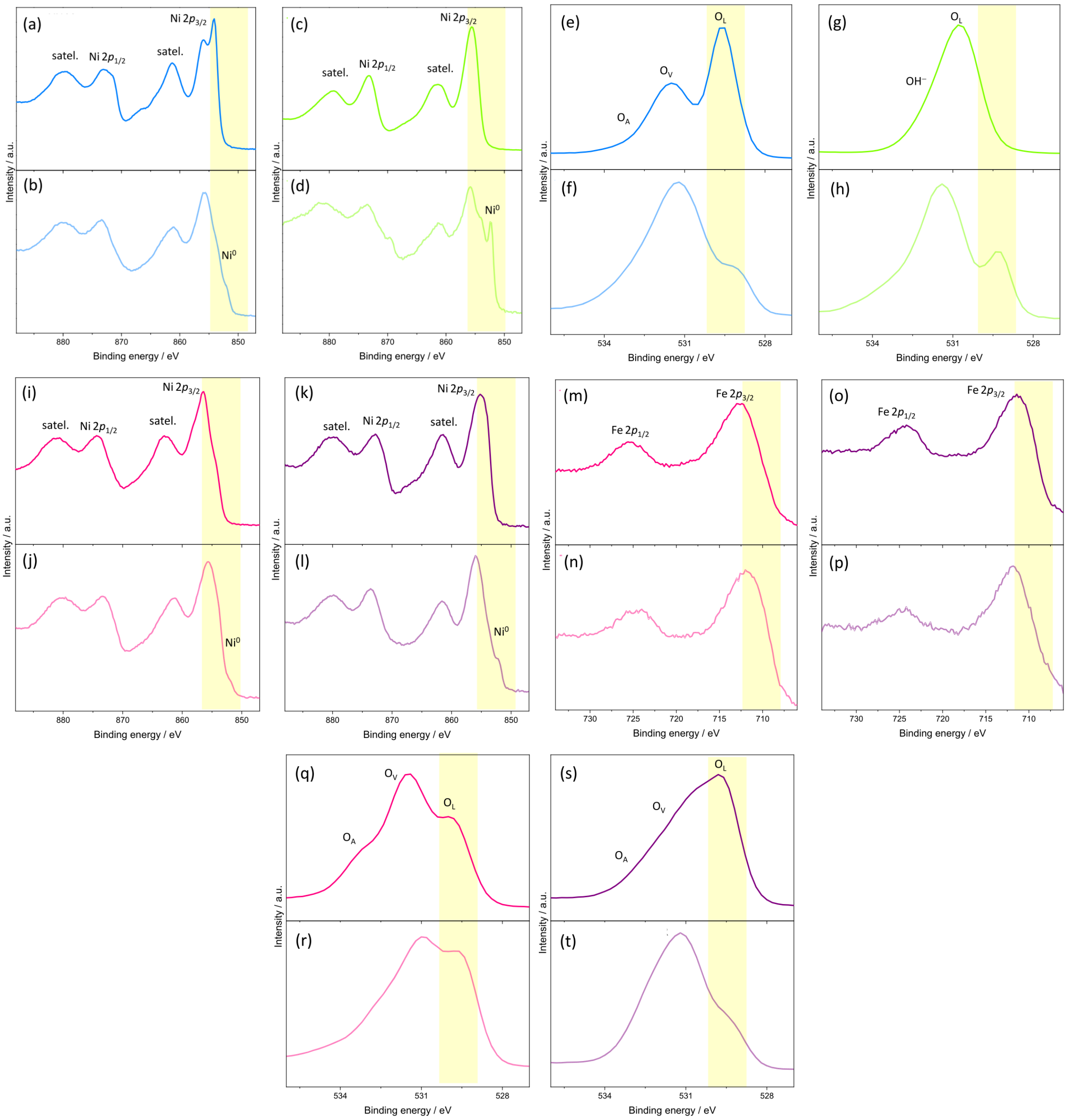

Figure S21. Comparison between HRXPS spectra of electrocatalysts in the (a,c,e,g,i,k,m,o,q,s) oxidized and (b,d,f,h,j,l,n,p,r,t) reduced form. Regions: (a–d,i–l) Ni 2*p*, (e–h,q–t) Ni 2*p* and (m–p) Fe 2*p*. The shown data refer to samples (a,e) Ni100, (b,f) Ni100R, (c,g) NiH100, (d,h) NiH100R, (i,m,q) Ni85Fe15_400, (j,n,r) Ni85Fe15_400R, (k,o,s) Ni85Fe15_800 and (l,p,t) Ni85Fe15_800R.

Electrochemical properties

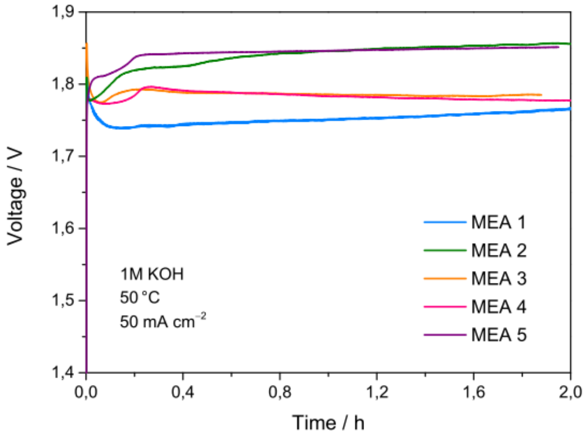

Figure S22. Conditioning at low current density (50 mA cm^{-2}) and 50°C.

Table S4. Performance and durability comparison of different membrane-electrode assemblies in similar AEMWE conditions.

**Anode (loading/mg cm^{-2})**	**Cathode (loading/mg cm^{-2})**	**Membrane**	**Ionomer**	**Electrolyte feed**	**T/°C**	**Current density/A cm^{-2} @cell voltage/V**	**Durability time/h @current density/A cm^{-2}**	**Ref.**
Ni85Co15_400 (2.5)	Ni85Co15_400R (3)	FAA-3-50 Fumatech	FAA-3	1M KOH	50	1@2.15	150@1	This work
IrO_2 (4)	Pt/C (0.4)	FAA-3-50 Fumatech	FAA-3-Br	1M KOH	70	1.5@1.9	-	[16]
CuCoOx (30)	Ni/(CeO_2–La_2O_3)/C (7.4)	A-201 (Tokuyama)	Alkaline I_2	1 wt% K_2CO_3	60	0.5@1.95	200@0.5	[89]
$NiFeO_x$ (2.5)	NiMo/KB (5)	FAA-3-50 Fumatech	FAA-3	1M KOH	50	1@1.8	2000@1	[30]
$NiFe_2O_4$ (2)	NiFeCo (2)	FAS-50	-	1M KOH	60	0.5@1.9	200@1	[90]
Ni-Fe-Ox (5)	Ni-Fe-Co (5)	PBI	Sustainion® XB-7	1M KOH	60	1@1.9	100@1	[91]
$NiCo_2O_4$ (2.5)	$NiFe_2O_4$ (2.5)	PPS/mTPN/ DABCO-Me	PSEBS-CM-DABCO	1M KOH	60	0.3@2	-	[92]
NiCu mixed oxide (5)	Ir black (3)	Fumapem-3-PE-30	Nafion ionomer	1M KOH	50	1.85@2	-	[27]

Table S5. Current densities, cell voltages and durability test times for AEM systems fed with 1 M KOH.

	**MEA 1**		**MEA 1**			**MEA 1**	**MEA 1**			**MEA 1**	
**Current density / A cm^{-2}**	0.2	0.4	0.2	0.8	0.6	1	0.6	0.8	0.6	0.4	0.6
**Cell Voltage / V**	1.94	2.18–2.2	2.05	2.06– 2.2	2.04– 2.2	2.15	2.12	2.18– 2.2	2.16– 2.2	2.16	2.17– 2.2
**Time / h**	42	6	69	26	17	146	22	68	20	96	20

Structural stability of the electrocatalyst

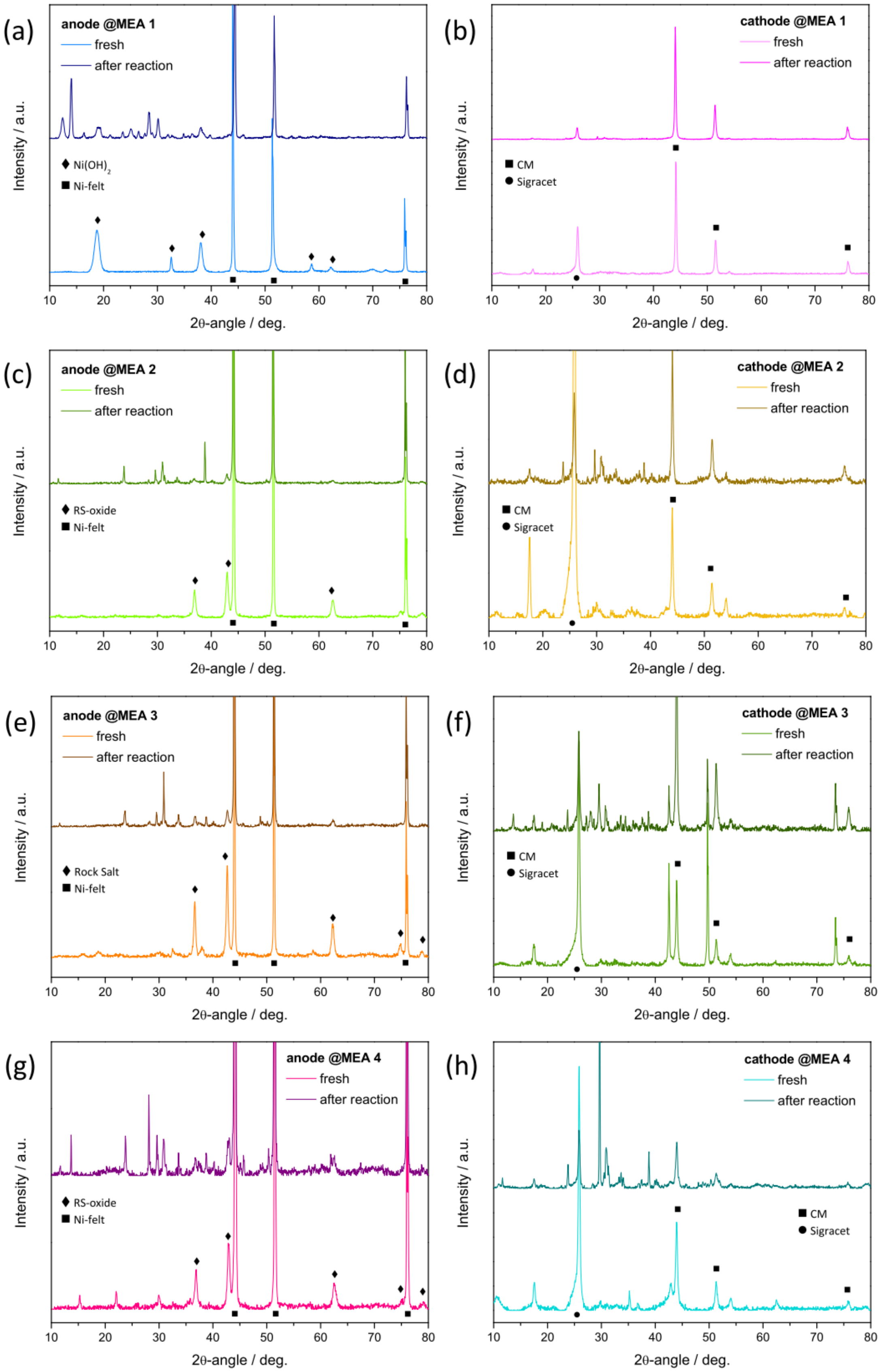

Figure S23. XRD patterns of (a,c,e,g) anodes and (b,d,f,h) cathodes utilized to fabricate (a,b) MEA 1, (c,d) MEA 2, (e,f) MEA 3 and (g,h) MEA 4 fresh and after reaction.
